# Assessment of clinical and microbiota responses to fecal microbial transplantation in adult horses with diarrhea

**DOI:** 10.1371/journal.pone.0244381

**Published:** 2021-01-14

**Authors:** Caroline A. McKinney, Daniela Bedenice, Ana P. Pacheco, Bruno C. M. Oliveira, Mary-Rose Paradis, Melissa Mazan, Giovanni Widmer

**Affiliations:** 1 Department of Clinical Sciences, Cummings School of Veterinary Medicine at Tufts University, North Grafton, Massachusetts, United States of America; 2 Department of Clinical Sciences, Carlson College of Veterinary Medicine at Oregon State University, Corvallis, Oregon, United States of America; 3 Faculdade de Medicina Veterinária, Universidade Estadual Paulista (UNESP), Araçatuba, Brazil; 4 Department of Infectious Diseases and Global Health, Cummings School of Veterinary Medicine at Tufts University, North Grafton, Massachusetts, United States of America; Midwestern University, UNITED STATES

## Abstract

**Background and aims:**

Fecal microbial transplantation (FMT) is empirically implemented in horses with colitis to facilitate resolution of diarrhea. The purpose of this study was to assess FMT as a clinical treatment and modulator of fecal microbiota in hospitalized horses with colitis.

**Methods:**

A total of 22 horses with moderate to severe diarrhea, consistent with a diagnosis of colitis, were enrolled at two referral hospitals (L1: n = 12; L2: n = 10). FMT was performed in all 12 patients on 3 consecutive days at L1, while treatment at L2 consisted of standard care without FMT. Manure was collected once daily for 4 days from the rectum in all colitis horses, prior to FMT for horses at L1, and from each manure sample used for FMT. Fecal samples from 10 clinically healthy control horses housed at L2, and 30 healthy horses located at 5 barns in regional proximity to L1 were also obtained to characterize the regional healthy equine microbiome. All fecal microbiota were analyzed using 16S amplicon sequencing.

**Results and conclusions:**

As expected, healthy horses at both locations showed a greater α-diversity and lower β-diversity compared to horses with colitis. The fecal microbiome of healthy horses clustered by location, with L1 horses showing a higher prevalence of Kiritimatiellaeota. Improved manure consistency (lower diarrhea score) was associated with a greater α-diversity in horses with colitis at both locations (L1: r = -0.385, P = 0.006; L2: r = -0.479, P = 0.002). Fecal transplant recipients demonstrated a greater overall reduction in diarrhea score (median: 4±3 grades), compared to untreated horses (median: 1.5±3 grades, P = 0.021), with a higher incidence in day-over-day improvement in diarrhea (22/36 (61%) vs. 10/28 (36%) instances, P = 0.011). When comparing microbiota of diseased horses at study conclusion to that of healthy controls, FMT-treated horses showed a lower mean UniFrac distance (0.53±0.27) than untreated horses (0.62±0.26, P<0.001), indicating greater normalization of the microbiome in FMT-treated patients.

## Introduction

Colitis continues to be a leading cause of critical illness in horses, with an estimated 25–35% mortality rate [1, 2]. This condition is accompanied by increased risks for severe complications (laminitis, coagulopathy, and cardiovascular dysfunction) which often require intensive care and prolonged hospital stays, placing a significant financial burden on owners and the equine industry [1].

Several studies indicate that the equine gastrointestinal microbiome of healthy horses has a significantly greater α-diversity compared to horses with colitis [3–5]. A diverse intestinal microbiota is thought to be essential for the maintenance of equine gastrointestinal health [6–8] whereas disruption of the resident commensal bacterial community of the gut (dysbiosis) may lead to colitis. Horses with colitis have highly variable bacterial communities (increased β-diversity) when compared to one another [3], perhaps reflecting the diverse causes of colitis. A specific etiology is not identified in the majority of horses with colitis. Without targeted therapies, clinicians cannot directly reverse the primary disease process that manifests in colitis, and instead rely upon supportive management to lessen the risk of life-threatening sequelae. Antibiotic treatment for colitis is controversial, and often contraindicated, as it could further derange the intestinal microbiota by eliminating commensal bacteria and allowing expansion of pathogenic species [6, 9]. Whether dysbiosis plays a causative role in this disease process or is a sequelae remains to be established [3, 10].

Reversing dysbiosis through fecal microbial transplantation (FMT), or the transfer of fecal microorganisms from a healthy donor horse into the intestinal tract of the recipient horse, is in early stages of clinical evaluation, but may provide a novel, directed therapeutic option [4, 11, 12]. Our recent work in a small number of geriatric horses with diarrhea showed improvement in diarrhea score, increased fecal microbiota α-diversity (greater number of bacterial taxa), and decreasing β-diversity in patients which responded to FMT [4]. Increased α-diversity following FMT has also been demonstrated in human patients with *C*. *difficile* infections [13, 14], ulcerative colitis [15] and infantile allergic colitis [16]. Several small equine studies have further suggested that FMT can result in clinical improvement of horses with acute and chronic diarrhea [8, 17] and normalization of fecal consistency in horses with increased free fecal water [18]. Resolution of diarrhea following FMT is best documented in people with refractory *Clostridium difficile* infections [13]. Similarly, FMT has been associated with a recovery of the gastrointestinal microbiome after antibiotic treatment in mice [19], and faster resolution of parvovirus-associated diarrhea in puppies compared to standard treatments [20]. Therefore, it may also represent a novel, cost-effective therapy for successful restoration of gut function in horses with colitis and resolution of diarrhea.

Our recent, preliminary work in geriatric horses with colitis [4] demonstrated that FMT responders developed an increasing relative abundance of Kiritimatiellaeota (formerly classified as Verrucomicrobia). These species are thought to be associated with colonic mucus production and promote the normalization of gut function [21]. Additionally, the fecal microbiota of treatment responders became phylogenetically more similar to that of their donor. Human FMT studies have also identified similar bacterial taxa in donors and recipients following microbiota transplantation [22]. The current study, completed within the ethical constraints governing the use of client-owned horses, was designed to expand our understanding of the clinical and fecal microbial profile response of horses with diarrhea. We hypothesized that FMT will allow for a more rapid re-establishment of a healthy, more diverse microbiome in horses with colitis that will resemble the donor microbiome, and improve diarrhea scores compared to control horses.

## Materials and methods

### Sample collection

The effect of FMT on the fecal microbiome of horses with diarrhea was evaluated using fecal samples of FMT-treated (Location 1, L1) and untreated horses (Location 2, L2) at two study sites. Both FMT donors (from L1) and healthy horses housed in regional proximity to locations 1 and 2 served as control horses. S1 Fig and S1 Table provides a study design overview.

This study was approved by the Institutional Animal Care and Use Committee and Clinical Studies Review Committee and met all requirements for ethical care and treatment of animals. Informed client consent was obtained for all client-owned horses.

#### Colitis horses

This prospective study included 22 adult horses treated at two separate referral hospitals (L1: n = 12; L2: n = 10), which either presented with or developed moderate to severe diarrhea (pudding to watery quality) while hospitalized, consistent with a diagnosis of colitis. Horses at both locations that developed diarrhea after presentation, did so within 48 hours of hospitalization, indicating that, though they were not yet producing diarrhea, their colitis may have contributed to their presenting complaint. All enrolled horses remained in the study unless another primary diagnosis, outside of colitis, was established. Fecal microbiota transplantation was performed in all 12 patients on 3 consecutive days at L1, while treatment of horses with diarrhea at L2 was based on standard care, without FMT. Horses with a history of reflux within 3 days prior to intended enrollment, recovering from colic surgery, or horses younger than two years of age were excluded. The obtained clinical data included: signalment, presenting complaint, predisposing factors such as antibiotic or non-steroidal anti-inflammatory therapy, recent long-distance travel, anesthesia, feed changes, prior enteral treatment with mineral oil, surfactants, or cathartics, duration of diarrhea, development of complications such as laminitis or thrombophlebitis, length of hospitalization, and outcome. Diarrhea frequency and quality was recorded every 6 h in colitis horses throughout the duration of the study. Diarrhea scoring was performed on a scale of 0–5 according to the following guidelines: 0—Normal, firm but moist balls of manure; 1—Soft-formed balls of manure that lose their form upon reaching the ground; 2—Pudding-consistency manure that still holds some shape; 3—Pudding-consistency manure that spreads out upon reaching the ground; 4—Watery manure with some formed pieces; 5—Watery manure without formed pieces.

Resolution of diarrhea was determined by the timepoint when manure quality reached a grade of 1 (soft-formed balls of manure that lose their form upon reaching the ground) or better, with no worsening of consistency throughout the remainder of the study period. Final outcome was characterized by survival (discharged alive) or non-survival (euthanasia or natural death).

#### Healthy horses at location 1—Controls and donors

Manure samples were collected at two time-points (two weeks apart) from 30 clinically healthy horses, located at 5 housing facilities in regional proximity to L1, as part of a preliminary study [4]. Three horses of this healthy cohort served as FMT donors. Complete diet history, medical history, and physical examination were obtained to ensure clinical health prior to fecal collection. Breed, age, body condition score, heart rate, respiratory rate, rectal temperature, attitude, and borborygmi were recorded. Exclusion criteria included any recent gastrointestinal illness (colic, diarrhea), transport, medical treatment, or dietary supplementation with probiotics. Up to 10 mL of feces were collected *per rectum* using a clean gloved hand at two separate time-points 2 weeks apart and stored at -80°C for subsequent analysis.

An Equine Diarrhea Polymerase Chain Reaction (PCR) panel for Coronavirus, *Clostridium difficile* toxins A and B, *Clostridium perfringens* antigens, *Lawsonia intracellularis*, *Neorickettsia risticii* and *Salmonella* sp. (Equine Diarrhea Panel, Research and Diagnostic Core Facility, University of California, Davis) and quantitative fecal egg count were confirmed to be negative for FMT donors. Prior to each FMT on 3 consecutive days, a 10-mL representative sample of the fecal material or suspension was obtained from the donor manure used as fecal transplant material for a single patient. These samples were visually evaluated to ensure that gross coloration and turbidity matched the suspension administered to the patient. All samples were stored and sequenced individually.

#### Healthy horses at location 2—Controls

Manure samples were collected at a single time-point from 10 clinically healthy control horses housed at L2, to compare the fecal microbiota between healthy horses and local patients with colitis. A single time-point was collected from this population, compared to the duplicate samples obtained from L1, due to funding limitations. Exclusion criteria and study methodologies were consistent between horses enrolled in L1 and L2. To ensure normal health prior to fecal collection, a complete diet history, medical history, and physical examination were obtained.

#### Clinical procedures

Fecal microbiota transplantation from a single donor horse was performed on 3 consecutive days for all patients with diarrhea at L1, using standard clinical techniques as previously published [4]. Briefly, 2.5 pounds of fresh manure was collected and mixed in 4 liters of lukewarm water either within a bouffant cap (serving as a standard sieve; McKesson 24-inch Disposable Bouffant Caps) or freely for 10 minutes. The mixture was subsequently strained (steel sieve, 2 mm hole diameter) and administered within 15 minutes of processing via nasogastric tube to the recipient horse. Following nasogastric intubation, recipient horses were evaluated for gastric reflux by creating a siphon with water and measuring the volume of water and gastric content returned compared to the water volume administered, equating to net reflux. No more than 2 L of water was lost into the stomach prior to administration of FMT, with < 2 L net reflux considered acceptable.

A 10-mL fecal sample was collected daily per rectum from all enrolled horses experiencing diarrhea (12 from L1; 10 from L2) for a total of 4 days. Two horses from L2 were discharged on day 3, which precluded sampling on day 4. For horses receiving FMT, fecal samples were collected prior to each microbial transplant (days 1–3) and 24 hours following the last FMT (day 4) (S1 Fig; S1 Table). All fecal samples were stored at -80°C in 3 mL aliquots. Blood was collected daily via intravenous catheter or venipuncture to measure packed cell volume (PCV), total solids (TS), and lactate.

At L1, all patients underwent *Salmonella* PCR testing (3 samples collected at ≥12 hour intervals), while a combined equine diarrhea PCR panel was only performed in 9/12 (75%) horses (Equine Diarrhea Panel, Research and Diagnostic Core Facility, University of California, Davis: Coronavirus; *Clostridium difficile* toxins A and B; *Clostridium perfringens* (CP) antigen, CP alpha toxin, CP beta toxin, CP beta2 toxin, CP cytotoxin (netF), CP enterotoxin; *Lawsonia intracellularis*; *Neorickettsia risticii* and *Salmonella*) to identify potential etiologies of their colitis.

All but one patient (VS) at L2 underwent equine diarrhea PCR panel analysis (Equine Diarrhea Panel, Oregon Veterinary Diagnostic Laboratory: including Coronavirus; *Lawsonia intracellularis*; *Neorickettsia risticii* and *Salmonella*) on one occasion to identify potential etiologies of their colitis. For both locations, submission of an equine diarrhea PCR panel was guided by clinical progression and owner financial investment.

### DNA extraction and 16S library preparation

DNA was extracted from 200 μl of thawed feces in a Qiacube instrument using the QIAamp PowerFecal DNA kit according to manufacturer’s instructions. Fecal DNA was eluted in 50 μl elution buffer and stored at -20°C. A 2-step PCR protocol described previously [23] was used to amplify and barcode the V1V2 16S rRNA region. A total of 106 barcoded amplicons were pooled in approximately equal proportion as assessed using a Qbit spectrophotometer. The size-selected library was sequenced 300-nucleotide single-end in an Illumina MiSeq instrument operated by the Tufts University genomics core facility (tucf.org). To control for technical variation, each library included duplicates of three randomly chosen samples. Duplicated amplicons were amplified from two DNA samples extracted in parallel from the same fecal samples. Each duplicated amplicon was tagged with a unique barcode. An amplicon generated from a synthetic bacterial population (BEI Resources, cat no. HM-782D) was also included in the library as quality control.

### Data analysis

Descriptive analyses of clinical data are presented as mean +/- standard deviation (SD) or median +/- interquartile range (IQR) or range. Univariate statistical analyses were based on the normality of data distribution (Shapiro-Wilk test) and utilized independent samples T-test, Mann-Whitney U or Chi-Square analyses to compare study groups. The analyses were performed using commercially available statistical software (IBM SPSS Statistics 26 and Sigmaplot v. 14), with an accepted significance level of P<0.05.

Including replicates and synthetic bacterial population, a total of 202 samples were included in the analyses. The total includes 97 samples analyzed previously [4] and deposited in NCBI’s sequence read archive under study accession number PRJEB32490. The new sequence data were deposited under accession numbers PRJEB32490 and PRJEB37702. FASTQ formatted sequence files were processed using programs in *mothur* [24] essentially as described [23]. An average of 118,413 raw sequences (n = 106 barcodes, SD = 30,297) were obtained per sample. The mean quality score for the entire library was 32.2. Each sample was randomly subsampled to 5,000 sequences. Sequences were curated by removing putative chimeras using uchime [25], by excluding sequences that did not align and sequences containing ambiguous base calls and homopolymers longer than 8 nucleotides. Of the initial 1,010,000 sequences, 884,082 sequences passed quality control and were used in downstream analyses.

Pairwise β-diversity between samples was quantified using weighted UniFrac [26]. Phylip-formatted distance matrices were imported into GenAlEx [27] and visualized using Principal Coordinate Analysis (PCoA). Sequences were clustered into Operational Taxonomic Units (OTUs) using the opti clustering method [28] and a 3% similarity cut-off. Constrained ordination analyses were performed to assess the association between independent variables, like location, diseases status, diarrhea score or treatment with antimicrobials and microbiota profile. Redundancy Analysis (RDA) and Canonical Correspondence Analysis (CCA) were performed in CANOCO [29]. Pseudo-F values are obtained by permuting the independent variables with respect to the OTU table. Program LEfSe [30] as implemented in *mothur* was used for Linear Discriminant Analysis (LDA).

Sequences were taxonomically classified using *classify*.*seqs* in *mothur*. Template and taxonomy files release 132 with 213,126 sequences x 50,000 columns were downloaded from SILVA [31]. A 70% probability cut-off was applied.

## Results

### Clinical data analyses of horses with colitis

#### FMT recipients with colitis (location 1)

Twelve client-owned horses, who were presented for colitis or developed diarrhea while hospitalized, received 3 consecutive daily fecal microbial transplants from a single donor horse. The horses’ clinical parameters, presenting complaints, hematologic characteristics, and diarrhea trends are specified in S2, S4 and S5 Tables. No horses at L1 received probiotics or prebiotics during the study period. All patients survived to discharge without significant complications, after 4 to 15 days of hospitalization (median 7 +/- 2.8 days).

Following etiological testing, one horse (CM) tested positive for Coronavirus and one for *Neorickettsia risticii* (AK). The latter was treated with Oxytetraycline (5.5 mg/kg intravenously every 12 hours; Zoetis, Kalamazoo, MI) during the study period, beginning 4 hours prior to his second fecal sampling. No other horses in this group were treated with systemic antimicrobials. Horse GH showed evidence of sand enteropathy based on abdominal radiographs and removal of enteric sand on rectal palpation. The remaining 9/12 (75%) horses, including two (DH and RC) that tested positive for *Clostridium perfringens* antigen, were diagnosed with undifferentiated colitis.

Three of the 12 enrolled horses (GH, TT, JG) were affected by Pituitary Pars Intermedia Dysfunction and one horse (DH) was diagnosed with mild lymphoplasmacytic inflammatory bowel disease, though no other chronic diseases or comorbidities were noted in the remaining population. Four horses enrolled at L1 presented to the hospital for suspected colitis while the remaining patients initially presented for abdominal pain. Fifteen horses were initially enrolled from L1, however 3 of the 15 horses were ultimately diagnosed with primary conditions unrelated to colitis (severe pneumonia, enterolithiasis, and lymphoma), and thus excluded from the study.

#### FMT-untreated horses with colitis (location 2)

Ten client-owned horses were presented for colitis or developed diarrhea while hospitalized at L2. The horses’ clinical parameters, presenting complaints, hematologic characteristics, and diarrhea trends are specified in S3–S5 Tables. Horses were hospitalized for 4 to 19 days (median 8.0 +/- 6.25), with 8/10 (80%) horses surviving to discharge. The two horses that were euthanized had significant morbidity associated with their disease, including thrombophlebitis, peritonitis (DC) and pulmonary thromboembolism (TM). Four horses enrolled at L2 received a commercialized prebiotic (Diamond V Original XPC) throughout the study entire period and one horse for the final 2 days of the study.

One horse (DC) tested positive for Salmonella at necropsy (ante-mortem testing was negative), two horses were positive for Coronavirus (PL and NE) and three for *Neorickettsia risticci* (LC, TM, MJ). Six of the ten horses enrolled at L2 presented to the hospital for suspected colitis, while the remaining four presented for abdominal pain (n = 3), or fever and lethargy (n = 1). Those positive for *Neorickettsia risticii* were treated with oxytetracycline (6.6 mg/kg intravenously every 12 hours; Zoetis, Kalamazoo, MI) throughout the study period beginning 24 hours prior to their first collected fecal sample. Another horse (BW) was treated with a combination of penicillin (22,000 IU/kg intravenously every 6 hours; penicillin G potassium; Athenex, Schaumburg, IL; Sandoz, Princeton, NJ) and gentamicin (6.6 mg/kg intravenously every 24 hours; VetOne, Boise, ID) throughout the study, beginning 24 hours prior to first sampling. In total, 4/10 (40%) of patients were diagnosed with undifferentiated colitis.

#### Statistical comparison of physical examination and hematological data between institutions

Signalment (age, breed, sex), body condition score and duration of diarrhea prior to enrollment were comparable between horses with colitis at the two study locations. However, median diarrhea scores were higher (more severe) at location 1 (FMT-treatment group) at the time of study enrollment (p = 0.05) (S6 Table). Similar etiological diagnoses were identified, apart from Salmonellosis which was only represented at location 2. At enrollment, median heart rate, packed cell volume, and serum lactate were statistically higher in horses at location 2 (S4 Table). Of these parameters, only heart rate was elevated outside the reference range. All other mean and median physical examination and laboratorial parameters were within reference range at both study sites upon study enrollment. At the end of the study period (Day 4), median lactate concentration was greater in the FMT-untreated group, but remained within reference range at both locations (L1: 0.82 +/- 0.3 vs L2: 1.4 +/- 0.4 mmol/L, p = 0.002). All other physical examination and hematological variables were comparable between study sites on the final day of sampling (S5 Table).

### Characterization of healthy horses

#### Control and donor horses at location 1

Healthy horses included 21 mares and 9 geldings, showing a median Body Condition Score (BCS) of 6 (+/- 2) and age of 12 years (+/-16), as previously reported [4]. Breeds included Morgans (10/30; 33.3%), Thoroughbreds (8/30; 26.7%), Quarter Horses (4/30; 13.3%), Paint Horses (3/30; 10%), an Appaloosa (1/30; 3.3%) and a grade horse (1/30; 3.3%). All horses were fed a median of 2.25% ± 0.25 body weight first cut hay per day, except for two horses which received 2% body weight second cut hay per day. The median daily concentrate intake was 4 pounds (+/- 4.8), with a median turnout time of 3 hours (+/- 6; S7 Table) on pasture.

Three horses, 2 geldings and 1 mare, housed at or nearby Location 1 were used as FMT donors for all colitis patients enrolled at study location 1. These donor horses were 6–12 years old (mean 9.3 +/- 1.8 years) with a median BCS of 6 (+/- 0.4) and included one Paint, Thoroughbred, and Quarter Horse. FMT donor horses received approximately 1–2% body weight of hay per day, 2–4 pounds of concentrate daily and were turned out in pasture for 3–14 hours per day.

#### Control horses at location 2

Ten healthy mares (median age: 19.5 +/- 4 years) housed at L2 under comparable management conditions, were sampled to serve as controls for horses with colitis at this location. Breeds included Quarter Horses (4), Paints (3), Thoroughbreds (2), and 1 grade horse. These horses received approximately 2% of their daily body weight as forage and were on-pasture for 9 hours per day (S7 Table). Body condition scores ranged from 5–7 (median 6 +/- 0.25) and did not differ from FMT donor scores at Location 1 (p = 0.47). Horses at both locations received similar quantities of hay per day (2% body weight, p = 0.4), although pasture turn-out was significantly longer at location 2 (L1: 3 +/- 6 hours vs L2: 9 hours, p = 0.001) and only one horse from location 2 was fed concentrate.

### Analysis of fecal microbiota

#### Quality control

The weighted UniFrac distance between three duplicated samples was 0.20, 0.01 and 0.24 (mean = 0.15). This compares to a mean of 0.50 for all 20,301 pairwise comparisons among the 202 samples included in the study. Phylum-level taxonomic classification of close to 130,000 sequences amplified from the BEI synthetic bacterial population gave the following results: Deinococcus-Thermus 4.3% (expected 5%), Actinobacteria 8.0% (expected 10%), Proteobacteria 26.9% (expected 30%), Firmicutes 40.0% (expected 50%), Bacteroidetes 9.2% (expected 5%), unclassified 3.2%, other classifications 8.4%.

#### Global analysis

**Fig 1:**
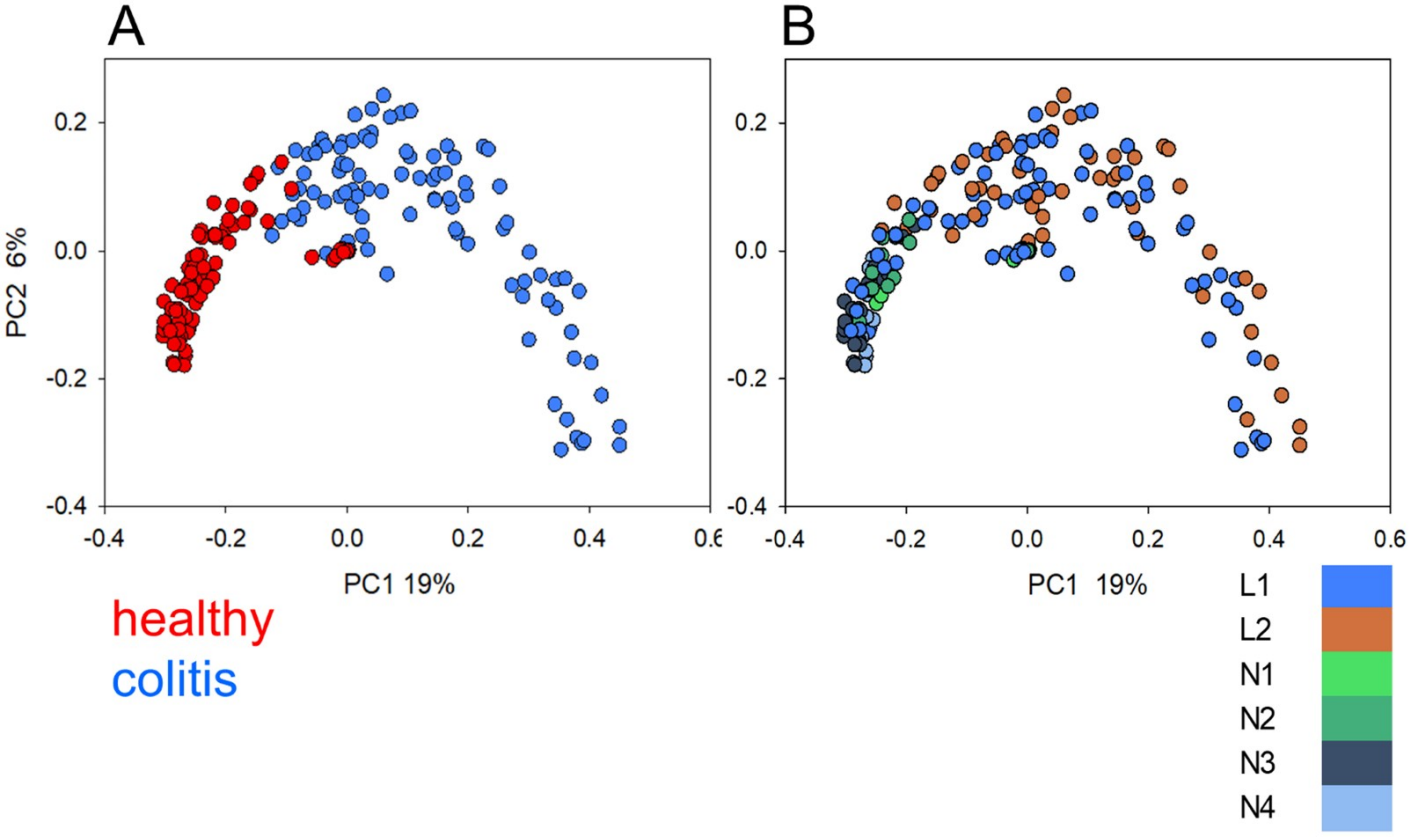
Principal Coordinate Analysis of 202 fecal microbiota from healthy and diseased horses. (A) Colors indicate disease status. (B) Colors indicate location. Locations on the East Coast of the United States are represented in blue and green, orange indicates West Coast location.

The β-diversity among 202 fecal microbiota was visualized using PCoA (Fig 1). When the plot is colored according to disease status, a relatively compact cluster of microbiota from healthy horses and more diversity among microbiota from diseased animals becomes apparent. This pattern was apparent regardless of the geographic origin of the samples. The effect of colitis on the microbiome is clearly stronger than the effect of geography.

As apparent in Fig 1, health status (FMT donor/healthy vs. FMT recipient/colitis) strongly influenced the microbiota. Canonical correlation analysis (CCA) using disease status as the only explanatory variable, indicates, as expected, a significant impact of colitis on the microbiota profile. This variable explains 5.2% of total OTU variation and is highly significant (pseudo-F = 11.0, P = 0.002). To assess the impact of location on the fecal microbiota, microbiota from diseased animals were excluded and pairwise weighted UniFrac distance values between 97 microbiota originating from healthy horses in 6 locations visualized by PCoA (S2 Fig). Included in this analysis are 27 samples from 3 L1 donor horses, one sample from each of 10 healthy controls from L2 and two samples each from N1, N2, N3 and N4 horses (S1 Table). The significance of clustering by location was tested with ANOSIM. L2 samples significantly clustered against all the other locations (R 0.33–0.50, p<0.001). Of the remaining 11 pairwise R values, only two, N2-N3 and L1-N3 were significant at p<0.001.

#### Diseased horses and donors

We examined whether the microbiota of L1 FMT recipients was distinct from that of L2 horses with colitis. This analysis was performed with CCA and included 85 samples from horses diagnosed with primary colitis at L1 and L2. The analysis revealed a significant association between location and microbiota profile. Although the fraction of OTU variation explained by location (and treatment) was relatively small (3.24%), the results of the permutation test indicate a significant effect of location (pseudo-F = 3.2, p = 0.001). Since the 85 samples originate from the entire duration of the study, the CCA results likely reflect the combined effect of treatment and location. To discriminate between the effect of these two variables, the analysis was repeated with microbiota from samples collected on day 1 of the study from diseased horses only. Twenty-two samples met this requirement, 10 from L2 and 12 from L1. This analysis revealed no effect of location (CCA, pseudo-F = 0.9, p = 0.94).

In contrast to the comparison of L1 and L2 horses with colitis, microbiota from healthy L1 donors and healthy L2 horses clearly differed by location. In an RDA with 37 samples, 10 from the same number of L2 horses and 27 samples from 3 L1 donor horses, location accounted for 13.7% of total variation and was significantly associated with the microbiota OTU profile (pseudo-F = 5.6, P = 0.002).

#### Diarrhea, treatment and microbiota

**Fig 2:**
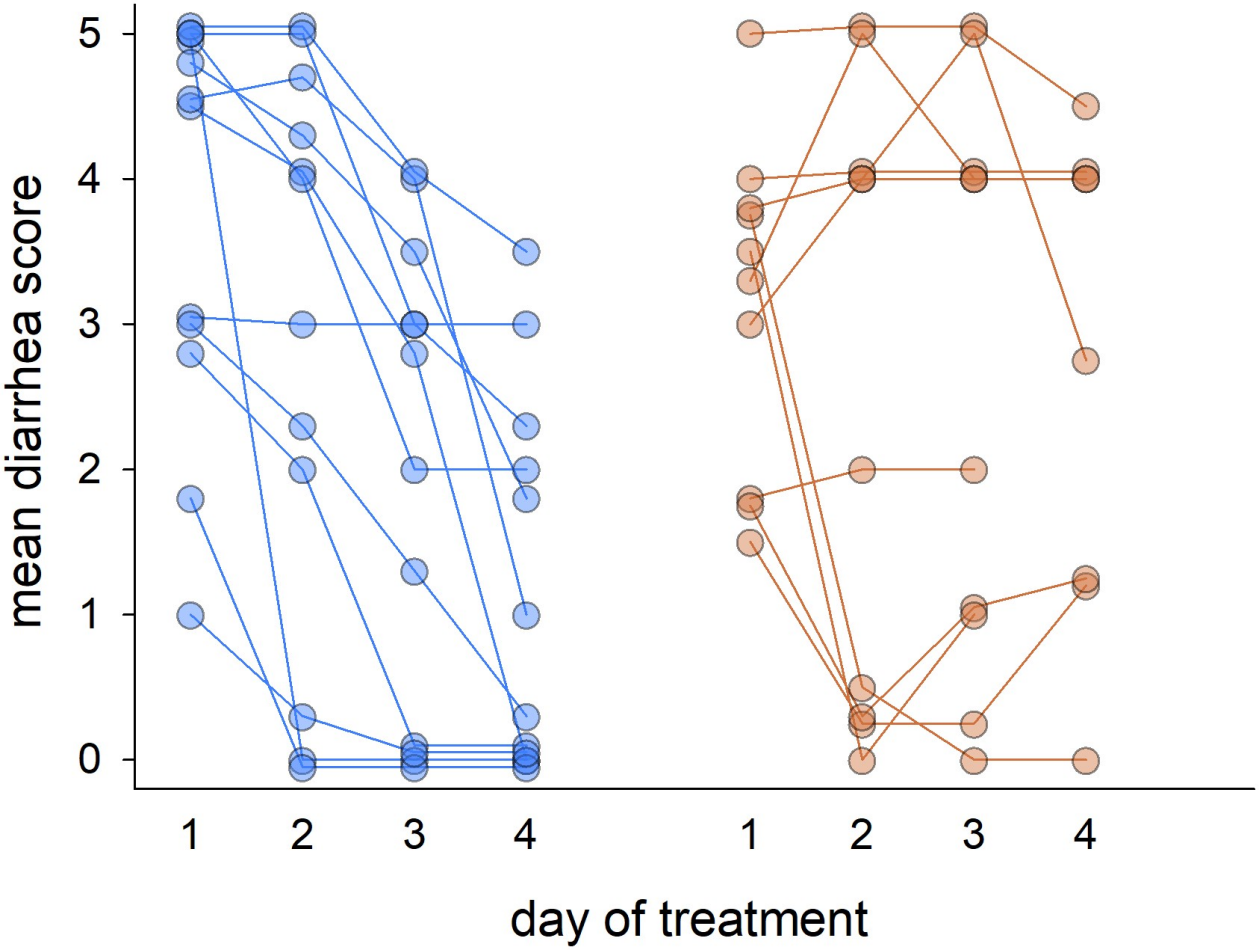
Mean diarrhea scores calculated from 4 daily observations. Blue, L1, n = 12; orange L2, n = 10. Overlapping data points were vertically offset by 0.05 to improve clarity.

A mean daily diarrhea score was obtained for 22 horses, 12 at L1 and 10 at L2, by averaging four daily scores obtained for all horses (Fig 2). We tested the effect of FMT and antimicrobial treatment using 3 metrics: day-over-day change in diarrhea score, mean diarrhea score on last recorded day, and difference between mean diarrhea score on first and last day.

Table 1 shows the day-over-day change in mean diarrhea score over 4 days assigned to 2 categories: improvement (decreasing score) or no improvement (no change or increasing score). The change is measured between 2 subsequent days. In 12 L1 horses, 22 instances of improving score out of 36 observations was recorded, whereas for L2 10 incidents of decreasing score (diarrhea improvement) out of 28 observations were noted (Table 1). The association between location and change in diarrhea score was significant (Fisher Exact Test: p = 0.038), consistent with a significant effect of FMT on diarrhea.

**Table 1:** Counts of day-over-day change in diarrhea score by location.

	L1 (FMT)	L2 (no FMT)
**Improvement**	22 (61%)	10 (36%)
**No improvement** (worse or no change)	14 (39%)	18 (64%)
**Total**	36	28

As a second measure of treatment effect, the median diarrhea score was compared between locations at each day of sampling (S6 Table). Although the median diarrhea score on the last day of sampling was lower at L1 (0; n = 12) as compared to L2 (1, n = 10) the difference was not statistically significant (Mann-Whitney, p = 0.069). Post-enrollment duration of diarrhea also did not differ between groups (p = 0.674). Finally, as a third measure of clinical improvement, the difference in diarrhea score between the first and last day of sampling was examined. The median diarrhea score of horses at L1 (FMT treatment group) improved by 4.0 scores (+/- 3, n = 12), more than twice the improvement observed at L2 (median = 1.5 +/- 3, n = 10; p = 0.021), consistent with a significant effect of FMT. As diarrhea is typically a manifestation of dysbiosis and loss of microbiota diversity, we examined whether such an association could be observed in the L1 and L2 fecal microbiota. A weak, but significant, negative correlation between diarrhea scores and microbiota Shannon diversity was identified in horses from both locations (S3 Fig). The Pearson correlation coefficient for L1 horses was -0.385 (n = 49, p = 0.006) and -0.479 (n = 41, p = 0.0015) for L2. While FMT resulted on average in a greater improvement in diarrhea score and more similarities between the microbiomes of donors and recipients (Fig 2, Table 3), improving diarrhea was associated with higher microbiota Shannon diversity irrespective of the treatment.

#### Fecal microbiota taxonomy

**Fig 3:**
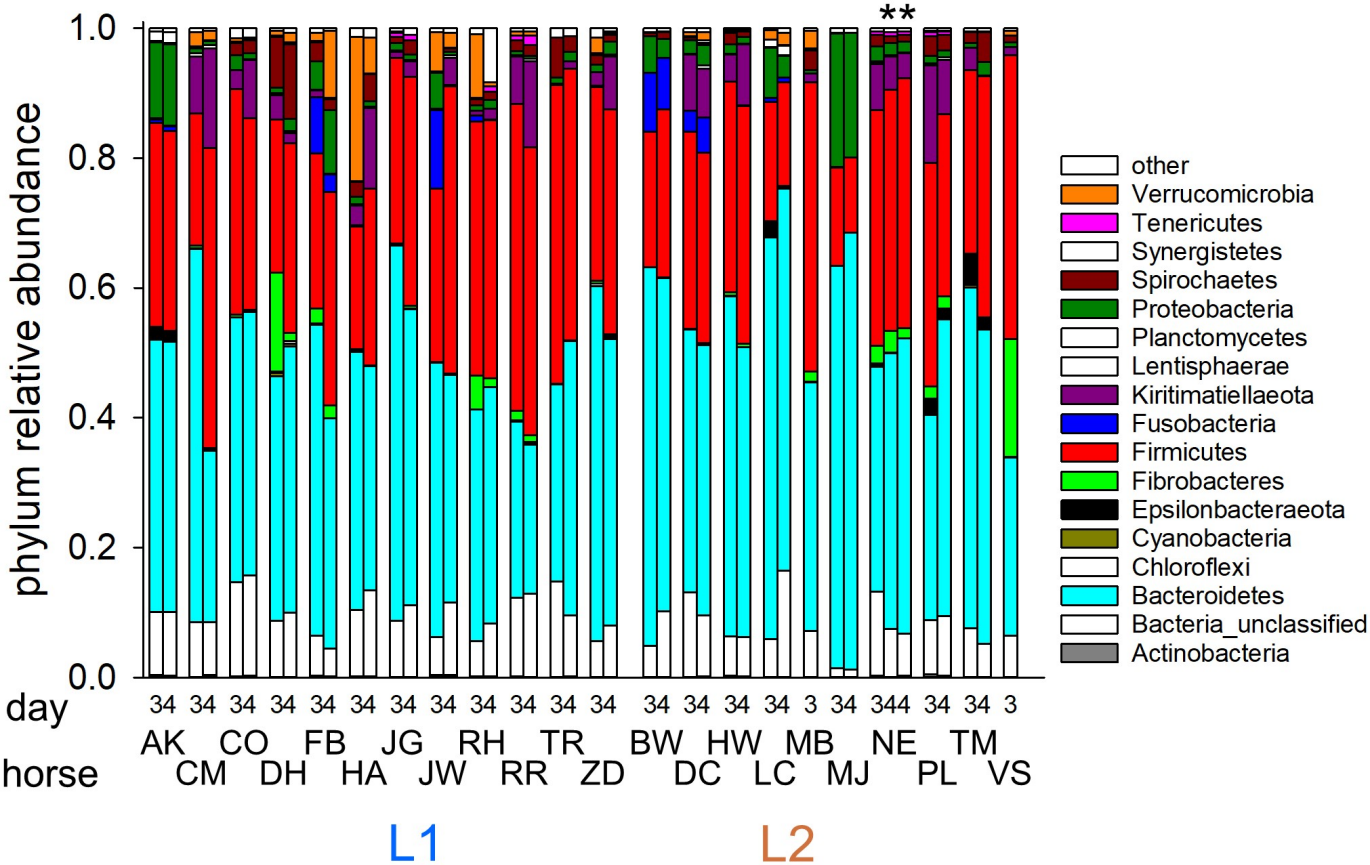
Relative abundance of microbiota phyla on last 2 days treatment for L2 colitis and L1 FMT recipients. Samples are grouped by location (left, L1; right L2). Within each location samples are grouped by horse. Bars 2 and 3 for horse NE are technical replicates as indicated by asterisks. Classification "other" includes sequences that were classified as Eukaryotes or Archaea.

Phylum-level relative abundance data for day 3 and 4 of treatment for 10 L1 and 12 L2 horses admitted for colitis are shown in Fig 3. The proportions of sequences assigned to major bacterial phyla and the variability between samples was similar to what was previously observed [4]. Bacteroidetes and Firmicutes were the most abundant taxa. The complete taxonomy of 202 samples is shown in S8 Table. LDA [30] was applied to investigate the differences between locations and treatment groups. The results are summarized in Table 2. The number of discriminative OTUs identified in these analyses reflects the taxonomic difference between the groups of samples being compared as well as the heterogeneity within each group of samples. Whereas only 34 significantly different OTUs were found when comparing microbiota from colitis horses at L1 and L2, the number of discriminative OTUs between healthy L1 and healthy L2 horses was 11 times greater (385). Given the extensive divergence between microbiota from diseased horses revealed by PCoA (Fig 1), this result indicates that colitis leads to very different dysbiotic states. This interpretation is consistent with the mean weighted UniFrac distances between healthy horses compared to those between diseased horses. Ignoring location, for the healthy microbiota the mean UniFrac distance is 0.33 (4437 comparisons, SD = 0.24), whereas distances between dysbiotic microbiota is almost double (0.63, 5054 comparisons, SD = 0.30). This level of heterogeneity among dysbiotic microbiota reduces the number of OTUs which reach significance in the L1 vs. OTU diseased horse comparison to only 34, as shown in Table 2. In contrast, when LDA is applied to the relatively homogeneous populations of L1 and L2 healthy microbiota, the number of significantly distinct OTUs is much larger, which is explained by the relative homogeneity of each group of healthy microbiota. The two cross-comparisons (L1 donor vs. L2 colitis; L1 recipient vs. L2 healthy) were not performed because the combination of two variables (location and disease) makes them less informative.

**Table 2:** Number of discriminative OTUs in four pairwise comparisons of fecal microbiota*.

		L2		L1	
		healthy	colitis	donor	recipient
L2	healthy		S9	S11	
	colitis	517			S10
L1	donor	385	-		3
	recipient	-	34	425	

* Lower triangle shows the number of discriminative OTUs; upper triangle indicates the table showing the corresponding taxonomic classifications.

To identify bacterial taxa that explain the differences between pairs of microbiota populations analyzed by LDA and shown in the lower triangle of Table 2, the taxonomic classification of discriminant OTUs was examined. Discriminant OTUs are those that significantly differ in relative abundance between the groups being compared. In this analysis the following groups are compared: healthy vs. diseased at each location, L1 donors vs. L2 healthy, L1 recipients vs. L2 diseased. In these 4 comparisons, the frequency of the phylum-level classification of OTUs identified by LDA was compared to the expected frequency based on the classification of all 3399 OTUs with 30 sequences or more found in the entire dataset (Table 3 and S9–S11 Tables). The expected frequencies shown in the column labeled "expected" are the total number of OTUs belonging to each phylum in the entire OTU table. This comparison is represented as a ratio in the tables in the column labelled "(donor + recipient)/expected". The values shown in columns "donor" and "recipient" are counts of OTUs identified by LDA as significantly over-represented in microbiota of healthy and diseased horses, respectively. These data enable a taxonomic interpretation of the data shown in Table 2. Specifically, they reveal bacterial taxa that define the difference between the four pairwise microbiota comparisons. In comparing L1 donor with L1 recipient (Table 3), 99 OTUs belonging to the phylum Kiritimatiellaeota were identified by LDA. These OTUs were all significantly overrepresented in the microbiota of healthy donor horses. Since, as indicated in column "expected", the taxonomic classifier identified 276 Kirimatiellaeota OTUs in the entire dataset, more than a third (99/276 = 36%) of this phylum’s OTUs differ significantly in relative abundance in the L1 donor *vs*. L1 recipient comparison. Significantly, all of these OTUs are overrepresented in the healthy donors. The analogous taxonomic analysis for the remaining three comparisons shown in Table 2 are presented in S9–S11 Tables. The apparent importance of Kirimatiellaeota in healthy *vs*. diseased comparisons is illustrated by the fact that 16% (47/296) of OTUs pertaining to this taxon were flagged by LDA in the L2 healthy *vs*. L2 diarrheic comparison (S9 Table). As in the L1 donor *vs*. recipient comparison, these Kiritimatiellaeota OTUs were overrepresented in the healthy microbiota, possibly an indication of the functional importance of this phylum in the healthy equine gut.

**Table 3:** Classification of OTUs significantly different in relative abundance between healthy L1 donors and L1 FMT recipients.

taxon	donor*	recip.*	expected**	(donor+recip)/expected***
Bacteria_unclassified	27	1	264	0.11
Bacteroidetes	140	15	1062	0.15
Cyanobacteria	6	0	28	0.21
Epsilonbacteraeota	0	1	3	0.33
Fibrobacteres	3	0	28	0.11
Firmicutes	86	15	1176	0.09
Kiritimatiellaeota	99	0	276	0.36
known_unclassified	1	0	3	0.33
Lentisphaerae	4	0	25	0.16
Proteobacteria	9	3	80	0.15
Spirochaetes	11	0	117	0.09
Tenericutes	2	0	24	0.08
Verrucomicrobia	0	2	8	0.25

* The total OTU count in columns "donor" and "recipient" is equal to the corresponding value shown in Table 2.

** Counts in column "expected" do not add up to 3399 because some phyla are not represented in the OTUs flagged by RDA.

*** Relative count of significantly different OTUs as a proportion of all 3399 OTUs in the dataset.

**Fig 4:**
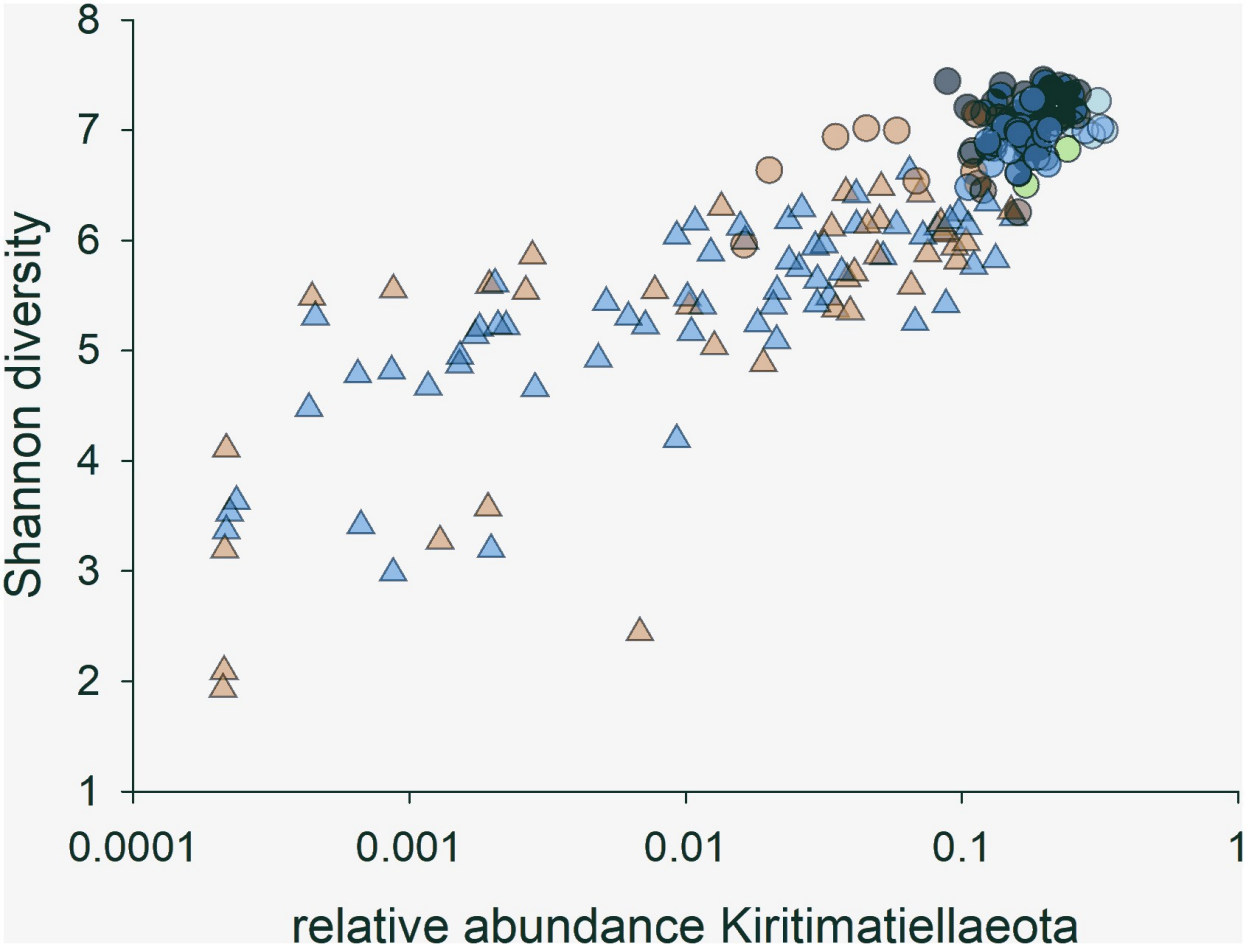
The relative abundance of Kiritimatiellaeota (genus WCHB1-41) strongly correlates with microbiota α-diversity. Triangles represent samples from diarrheic horses and circles healthy horses. Colors indicate location, where orange is for L2 and green-blue colors represent the same east coast locations as shown in Fig 1, i.e., L1 and N1-N4.

The presence of numerous Kirimatiellaeota OTUs among those identified by LDA as defining the difference between healthy and dysbiotic microbiota is of interest to understanding the pathogenesis of equine colitis. In light of the results presented in Table 3, S9 and S11 Tables, variables associated with Kirimatiellaeota abundance were further investigated. In average, Kirimatiellaeota sequences were 5 times more abundant in healthy microbiota as compared to dysbiotic microbiota (Mann-Whitney U Statistic = 301.0, p<0.001). The vast majority of Kiritimatielleoata sequences (mean = 98.6%, SD = 0.05) were classified as genus WCHB1-41 [32]. As shown in Fig 4, a strong positive correlation (r = 0.75, n = 202, p = 7.2e-38) between Shannon diversity and the log of the relative Kirimatiellaeota abundance was found. In the 4 microbiota samples with the highest proportion of Kiritimatiellaeota, WCHB1-41 sequences average 31% (range 30.0–32.8) of all sequences. Such a high proportion of identical sequences is typically associated with low α-diversity. Fig 4 shows that the opposite is the case; a high relative abundance of WCHB1-41 sequences strongly correlates with high Shannon diversity. To further investigate this unexpected outcome, rank-abundance curves were plotted for the four samples with the highest WCHB1-41 relative abundance and four samples devoid of such sequences (S4 Fig). This analysis shows that microbiota from healthy horses not only are more diverse, but are also more even. These results indicate that the relative abundance of WCHB1-41 may be a marker of a taxonomically diverse microbiota, even if this taxon is highly abundant. Excluding 60 samples which were obtained from horses kept at locations other than L1 and L2 and were not part of the FMT study (see S1 Table), WCHB1-41 sequences were particularly abundant in the 3 L1 donors (S5 Fig). Comparing healthy L1 and L2 horses, the mean relative WCHB1-41 abundance among healthy L1 microbiota samples averaged 0.16 (n = 23) against 0.07 (n = 10) for healthy L2 horses (p = 6.6e-9).

As previously reported, FMT can reduce β-diversity between donor and recipient [4]. Since the present study included diarrheic horses who were not treated with FMT, the β-diversity between healthy controls and conventionally treated horses, and between FMT donors and matching recipients, was compared. Using the weighted UniFrac distance as measure of β-diversity, pairwise distances between donor and recipient on FMT days 3 and 4 were compared to the distances between healthy and diseased L2 horses. This analysis revealed a reduction in β-diversity between FMT donors and recipients (mean UniFrac distance = 0.53, SD = 0.27) as compared to L2 healthy *vs*. L2 diseased (mean = 0.62, SD = 0.26). This difference is statistically significant (Mann-Whitney Rank Sum Test p<0.001). Also in agreement with the known effect of antibiotics on the fecal microbiota in other species [33], Shannon diversity for L1 and L2 horses classified in three treatment categories (no antimicrobials, KPen and Oxytetracycline; S6 Fig) shows a reduction in α-diversity in response to antimicrobial treatment (Kruskal-Wallis One Way Analysis of Variance on Ranks; H = 36.470, 2 d.f., p<0.001).

Having observed differences in the microbiota profile between healthy L1 and L2 horses (S1 and S4 Figs), we examined to what extent these differences could potentially be associated with diet. We used RDA to test whether concentrate, measured in kg/day, and pasture turnout (h/day) were significantly associated with OTU profile, where the microbiota profile is based in this analysis on 580 non-zero OTUs. This analysis included 43 horses (S1 Table). Because multiple samples were sequenced for each horse, mean OTU abundance was calculated by averaging the relative OTU abundance over all samples collected from each horse. Concentrate amount and pasture turnout were defined as independent variables, whereas age and BCS were defined as covariates. This analysis showed that kg/day concentrate was significantly associated with the OTU profile (pseudo-F = 2.1, p = 0.002) and explains 6.0% of OTU variability. The availability of microbiota data from healthy horses at locations N1-N4 strengthened the analysis in showing that the effect of concentrate in the diet is likely to be independent of the location. However, since 9/10 L2 horses were not fed any concentrate, this conclusion remains tentative.

## Discussion

In this study, diarrhea severity improved more quickly in horses receiving FMT for 3 consecutive days compared to FMT-untreated horses. The FMT-treated group had more severe diarrhea on enrollment, which may explain why the overall duration of diarrhea and final diarrhea scores did not differ between the two patient groups. When comparing the microbiota of diseased horses at study conclusion to that of healthy controls, FMT-treated horses showed a lower mean UniFrac distance to their donors than untreated horses did to their healthy controls, indicating greater normalization of the microbiome in FMT-treated patients. Normalization of the microbiome is critical to attaining gastrointestinal health, as the microbiome plays an important role in digestion, development of the gut immune system through mucus production and anti-inflammatory signaling, and maintaining metabolic function [34, 35].

Enrolled colitis horses were comparable between locations based on their signalment (age, gender, and breed), body condition score, and mean duration of diarrhea prior to enrollment (S4 Table). At the time of enrollment, patients from L2 had higher markers of volume depletion (heart rate, packed cell volume, lactate) while patients at L1 had significantly worse diarrhea scores, suggesting a more liquid diarrhea quality, despite a lesser degree of systemic volume depletion in the colitis group at L1. Causes for this discrepancy may include the subjective nature of diarrhea scoring and possible difference between evaluators at location 1 and 2, inability to quantify amount of fluid loss and volume of diarrhea as well as inability to quantify amount of fluid loss into the colon. Overall, the horses’ illness severity was deemed similar, justifying comparison of treatment effects between locations.

The inclusion of horses from two locations to investigate the impact of FMT on colitis represents a significant improvement over our previous study [4], as horses at location L2 did not receive any fecal transplants. Unaffected horses from each location were included to provide location-specific healthy microbiota profiles for comparison to those of horses with colitis. These groups were comparable in vital parameters, body condition score, age, and breed though differed in sex distribution, with only mares included in the healthy control group from L2. Previous works have not demonstrated sex-dependent differences in the equine fecal microbiome [4, 7, 36]. At the same time, the study design is not perfect because location represents an additional variable which cannot be controlled. The limitations of the inclusion of L1 and L2 horses arises primarily from the fact that healthy L2 horses, in contrast to healthy L1 horses, were not fed any significant amount of concentrate. The effect of location and treatment on the fecal microbiota cannot be separated, as these variables are 100% correlated. The difference in the microbiota profile observed between healthy L1 and L2 horses illustrates that such concerns are justified.

Etiological causes of colitis were similar between colitis groups, with Equine Coronavirus (L1: 2; L2: 2) and *Neorickettsia risticii* (L1: 1; L2: 3) identified in both locations. Salmonellosis was only diagnosed in one FMT-untreated horse on post-mortem assessment. As treatment for *Neorickettsia risiticii* requires antimicrobial therapy (tetracyclines), more horses at location 2 (3) received antimicrobials than at location 1 (1). Additionally, one horse from location 2 was treated with penicillin G potassium and gentamicin due to concerns of generalized sepsis. Antimicrobials have been previously demonstrated to have a significant impact on the equine microbiome in health [37, 38] and in disease [4]. As more horses received antimicrobials at L2, this treatment may have contributed to differences between the microbiome changes in this location compared to L1.

In contrast to the microbiota in healthy horses, the fecal microbiota of diseased horses did not segregate by location. Based on Canonical correlation analyses comparing the microbiota of samples collected from horses with colitis on day 1 of the study, we concluded that location alone is unlikely to explain the distinct OTU profiles in horses treated for colitis at L1 and L2. This interpretation is consistent with Fig 1B which shows no evidence of segregation by location in diseased horses. This observation, together with the large β-diversity values between fecal microbiota of diseased horses, illustrates the fact that different etiologies of colitis can lead to very different dysbiotic states. The greater reduction in diarrhea severity of horses receiving microbial transplants in this study complements our previous work [4]. Though the mean duration of diarrhea prior to the initiation of sampling was statistically comparable between groups, two of the horses at L1 had chronic diarrhea (>5 years), whereas all L2 horses showed a more acute onset of colitis. Both chronically affected horses resolved their diarrhea prior to hospital discharge. One of these horses (GH) was affected by sand enteropathy and represented to the hospital approximately 18 months later with evidence of re-accumulation of sand and recurrence of diarrhea. The second horse (JG) has maintained formed manure since discharge from the hospital, with an 18 months follow-up period at the time of manuscript submission. This observation is consistent with anecdotal reports of improved fecal consistency in equine colitis [8, 11, 17] and reduction of excessive free fecal water after FMT treatment [18]. In addition to improved manure consistency reported in a small group of horses with antibiotic-induced or undifferentiated colitis [17], FMT has also been associated with a more rapid resolution of fever when administered to horses with acute diarrhea following exploratory celiotomy compared to untreated horses [39]. Given that clinical variables improved in both the FMT-treated and untreated groups in the current study, the relationship between FMT and alterations in vital parameters could not be established in this study. However, the greater overall improvement in manure consistency over 4 days (P = 0.03) and higher incidence in day-over-day diarrhea improvement (p = 0.038) in FMT-treated horses, suggest clinical efficacy of FMT. A higher diarrhea score was associated with a lower microbiota *α*-diversity in horses with colitis at both study sites as previously observed [4]. Similarly, diarrheic horses at both locations showed a significantly lower abundance of Kiritimatiellaeota bacteria when compared to healthy L1 controls. Duration of diarrhea was not affected by FMT and assessment of mortality would require evaluation of a larger sample size.

A significantly greater relative abundance of Kiritimatiellaeota was found in healthy L1 and N1-N4 horses. Apart from this taxon, healthy horses shared similar predominant phyla including Firmicutes and Bacteroides, as previously identified by others [3, 40]. Higher volume concentrate feeding has been associated with increased abundance of RFN20 (family Erysipelotrichacea of Firmicutes phylum) and Bacillus-Lactobacillus-Streptococcus (BLS) group bacteria in the equine microbiome [34, 41]. Diet has not yet been associated with changes in the relative abundance of Kiritimatiellaeota or Verrucomicrobia, as this phylum was previously named. It is, therefore, unclear if this management factor contributed to the observed difference in Kiritimatiellaeota relative abundance between locations.

The fecal microbiota of equine species has been investigated in several laboratories, [7, 40, 42, 43]. This research has focused on the effect on the fecal microbiota of several variables, like diet [44], age and body condition [40, 42], GI tract anatomy [7] and host taxonomy [21]. Because of differences in wet-lab and bioinformatics methods, it is often difficult to compare results from different laboratories other than at a superficial level. Phylum level taxonomies reported in various studies broadly agree in the relative abundance of the main phyla Bacteroidetes, Firmicutes and less abundant taxa like Verrucomicrobia. The latter phylum includes anaerobic species of significant functional interest. For instance, the species *Akkermansia muciniphila* [45] is commonly detected in the healthy colon of various mammalian species [46–48]. The functional importance to the host is their affinity for the mucus layer of the GI tract and the fact that they do not metabolize molecules originating from the diet or excreted by other colon-dwelling bacteria. Instead, these bacteria degrade mucins from the mucus layer. Because the mucus layer is a hallmark of a healthy gut [49], the extensive variation in the relative abundance of Verrucomicrobia is potentially of interest to understanding the interaction between the equine colonic microbiota and the host, and mechanisms leading to dysbiosis. Confirming the potential functional relevance of these bacteria, in our previous study [4] we observed a negative association between the relative abundance of Verrucomicrobia sequences and severity of diarrhea. The taxonomies described in our previous publication were based on version 128 SILVA SSU database, which does not feature the phylum Kiritimatiellaeota [50]. Consequently, sequences previously assigned to the phylum Verrucomicrobia are classified in the present study as Kiritimatiellaeota.

In some analyses of equine microbiota, Verrucomicrobia are among the most abundant phyla [7, 51]. This phylum was also found to be abundantly represented in an equine core microbiome shared by multiple equine species and subspecies [21]. Other studies did not detect any [40] or relatively few Verrucomicrobia sequences [42]. These striking differences appear to be unrelated to the 16S variable region sequenced; Morrison et al. [40] sequenced a very similar 16S gene region as in the present study, with the upstream primer presumably being identical or very similar as the 27F primer used here, yet detected no or few Verrucomicrobia sequences. Differences in the reference taxonomy may have impacted the reported taxonomies, as Morrison et al. used an unspecified version of the RDP reference. The new perspective on these anaerobic bacteria revealed in the present study is the effect of colitis on their abundance. Although the present work, as well as previously reported observations [4], consistently show the depletion of Kiritimatiellaeota genus WCHB1-41 in horses with diarrhea, the 4-day time span analyzed here is insufficient to assess whether WCHB1-41 bacteria respond to FMT differently than to conventional treatments. Because these bacteria likely populate the mucus layer, it is conceivable that their depletion from the microbiota of diseased horses is a result of the thinning of the mucus layer, which has been observed in humans and in animal models [49]. Of particular interest would be to examine whether WCHB-1 abundance promotes recovery from colitis or is simply a consequence of the re-establishment of anaerobic conditions following an episode of diarrhea [52].

In summary, this study supports the use of FMT as a treatment to reduce diarrhea severity in horses with colitis and to improve microbiome diversity. Diarrheic horses undergoing serial FMT showed a greater improvement in diarrhea severity compared to non-treated horses, and their microbiome became more phylogenetically similar to that of their donors. While these results support the utility of FMT, as previously proposed [4], further studies utilizing a larger sample size and controlled etiologies and management of colitis are needed to better establish FMT treatment efficacy. Evidence of intestinal colonization by transplanted microbiota support the need to identify novel probiotics to accelerate re-establishment of a healthy microbiome. Additionally, with increasing support for the use of FMT, a need to further investigate the treatment’s mechanism of action, to standardize and streamline administration protocols, develop veterinary stool banks, and explore effective storage options, may expand the accessibility of this treatment to equine practitioners.

## Supporting information

S1 Fig Study timeline overview. (TIF)

S2 Fig Principal Coordinate Analysis of 97 samples from 22 healthy horses and 6 locations reveals effect of location. (TIF)

S3 Fig Mean diarrhea score *vs*. microbiota Shannon diversity. (A) 12 L1 horses. (B) 10 L2 horses. Duplicated samples are indicated with matching triangles.
(TIF)

S4 Fig Genus-level log-linear rank-abundance plot of four microbiota samples. The red symbols represent four microbiota with the highest proportion of Kiritimatiellaeoata genus WCHB1-41 among the 202 samples analyzed. Four WCHB1-41-negative microbiota are represented with blue symbols. Each circle represents the abundance of a genus. Genera are ranked from left-to-right in order of decreasing relative abundance. Vertically aligned symbols have the same rank but do not necessarily represent the same taxon. The graph shows that a high proportion of WCHB1-41 in the healthy microbiota was not associated low diversity (evenness), as indicated by a more even distribution of genus relative abundance in healthy microbiota as compared to the diseased samples.
(TIF)

S5 Fig Relative abundance of Kiritimatiellaeota WCHB1-41 by day of treatment and location. Each circle represents a sample obtained on the day shown. Note the large difference between L1 donors (blue) and L2 healthy controls (orange).
(TIF)

S6 Fig Antibiotics decrease microbiota α-diversity. Four samples in the KPen group came from one horse, 16 samples came from 4 horses treated with oxytetracycline and 53 samples are from 13 horses which were not treated with antimicrobials.
(TIF)

S1 Table Procedure and sample collection timeline. (DOCX)

S2 Table Historical information of horses with diarrhea (colitis) at location 1 receiving FMT. (DOCX)

S3 Table Historical information of horses with diarrhea (colitis) at location 2 not receiving FMT. (DOCX)

S4 Table Clinical enrollment parameters (mean +/- Std Dev) of horses with colitis. (DOCX)

S5 Table Final clinical parameters (mean +/-Std Dev) of horses with colitis upon study completion. (DOCX)

S6 Table Median diarrhea scores (+/- IQR) of horses with colitis over time. (DOCX)

S7 Table Healthy donor and control horses’ phenotype, diet, and location (L). (DOCX)

S8 Table Taxonomy of 202 microbiota. (XLSX)

S9 Table Classification of OTUs significantly different in relative abundance between L2 healthy and L2 diarrheic horses. (DOCX)

S10 Table Classification of OTUs significantly different in relative abundance between diseased L1 and L2 horses. (DOCX)

S11 Table Classification of OTUs significantly different in relative abundance between healthy L1 donors and healthy L2 horses. (DOCX)
