# Supplementary figures and images for: Assessment of clinical and microbiota responses to fecal microbial transplantation in adult horses with diarrhea
Source: PLoS One. 2021 Jan 14;16(1):e0244381. doi: 10.1371/journal.pone.0244381 (PMC7808643; doi:10.1371/journal.pone.0244381)

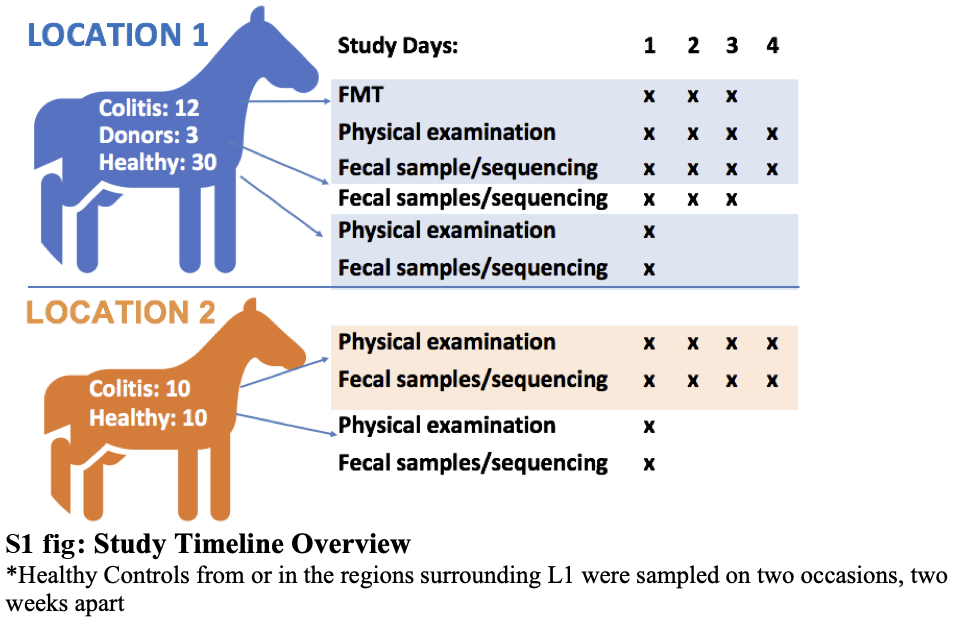

Supplement: S1 Fig — (TIF) [file pone.0244381.s001.tif]

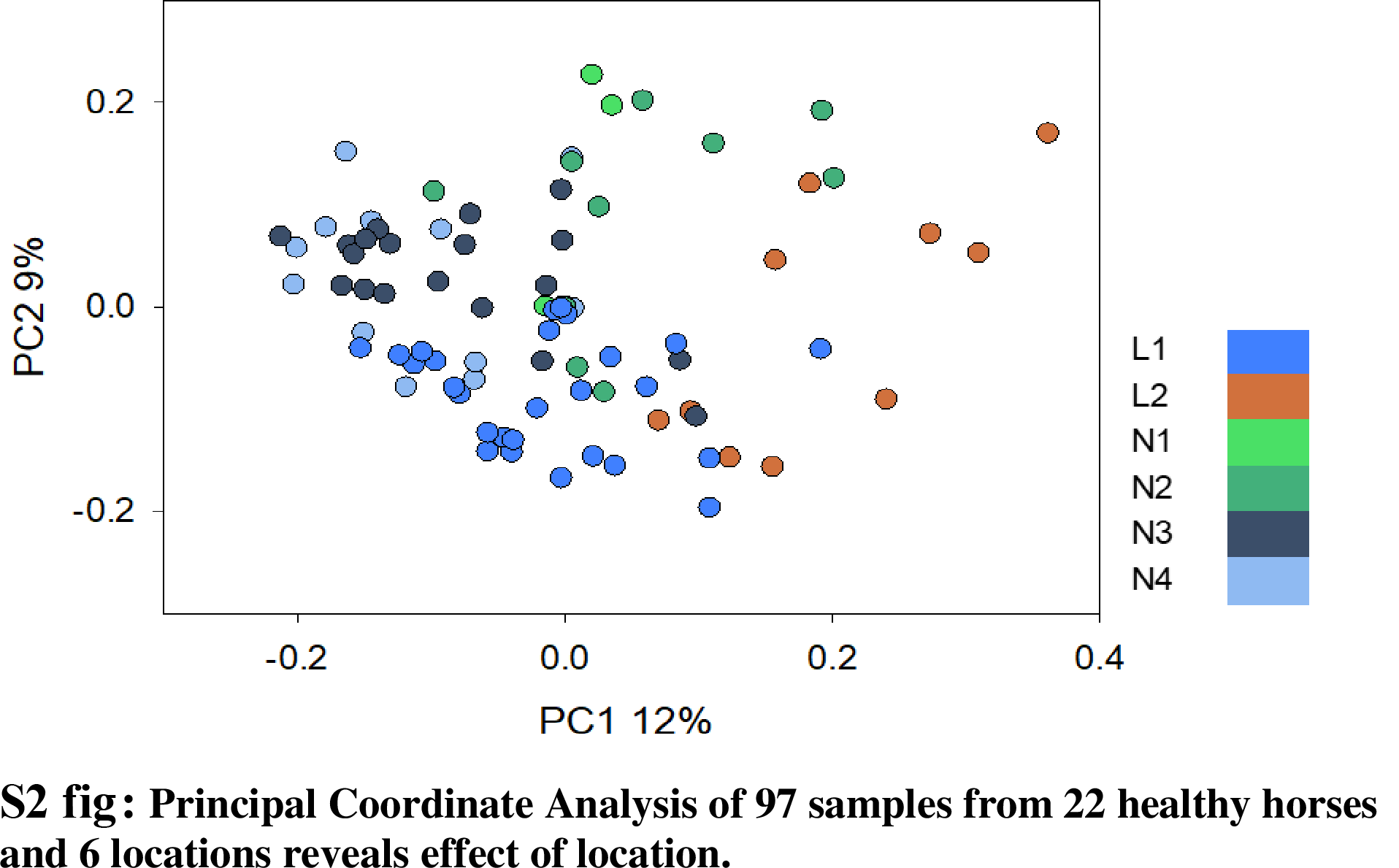

Supplement: S2 Fig — (TIF) [file pone.0244381.s002.tif]

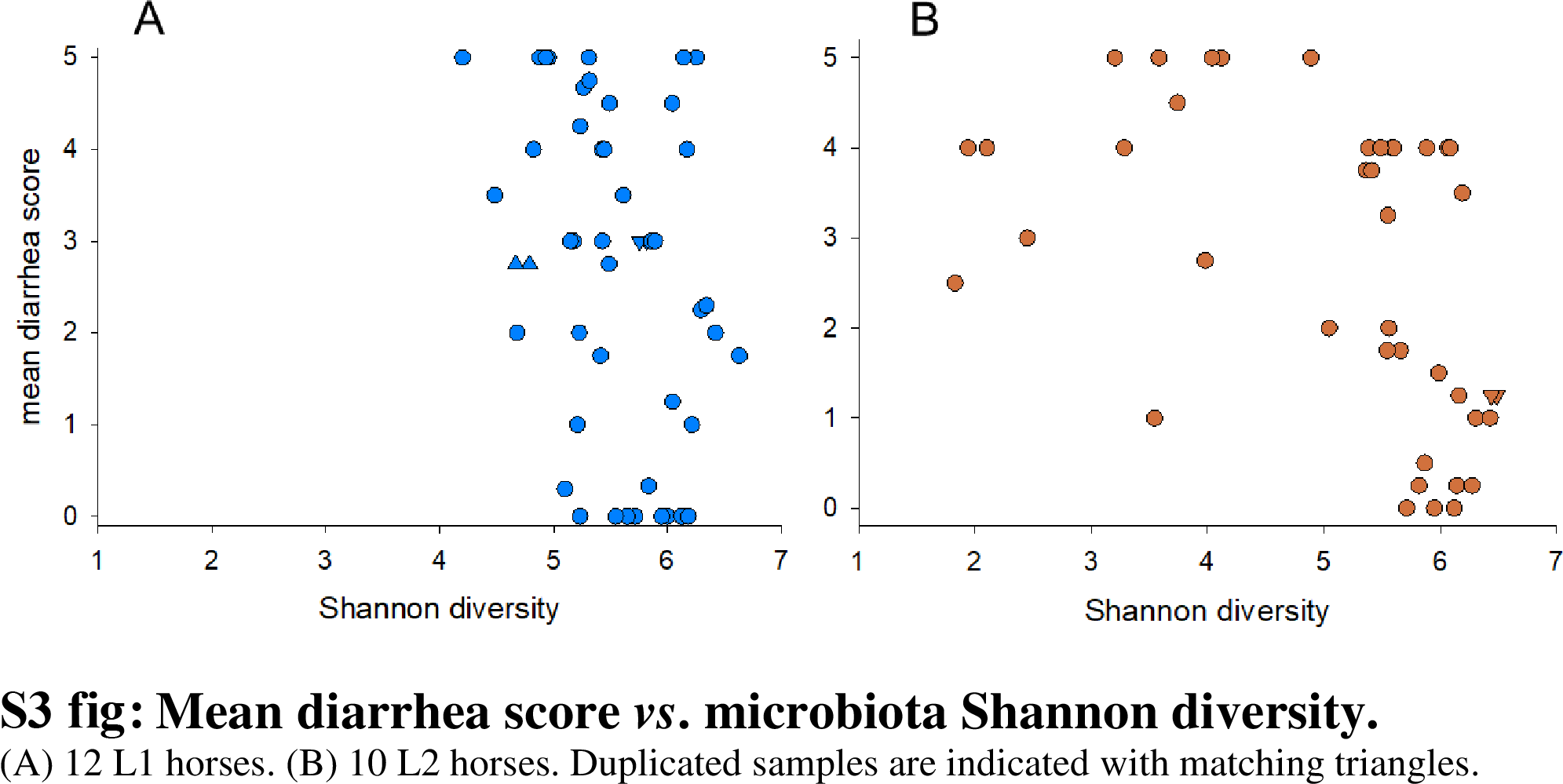

Supplement: S3 Fig — (A) 12 L1 horses. (B) 10 L2 horses. Duplicated samples are indicated with matching triangles. (TIF) [file pone.0244381.s003.tif]

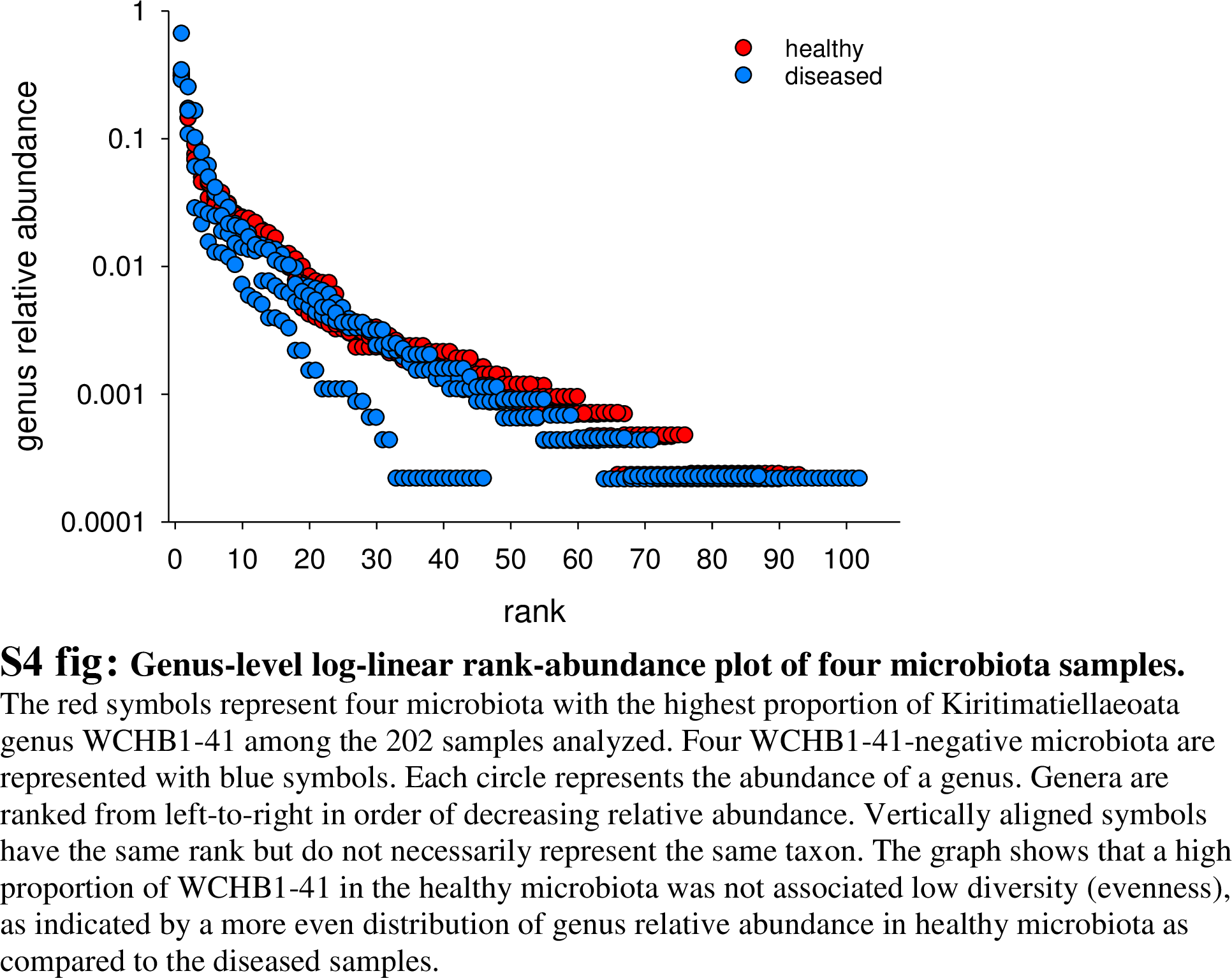

Supplement: S4 Fig — The red symbols represent four microbiota with the highest proportion of Kiritimatiellaeoata genus WCHB1-41 among the 202 samples analyzed. Four WCHB1-41-negative microbiota are represented with blue symbols. Each circle represents the abundance of a genus. Genera are ranked from left-to-right in order of decreasing relative abundance. Vertically aligned symbols have the same rank but do not necessarily represent the same taxon. The graph shows that a high proportion of WCHB1-41 in the healthy microbiota was not associated low diversity (evenness), as indicated by a more even distribution of genus relative abundance in healthy microbiota as compared to the diseased samples. (TIF) [file pone.0244381.s004.tif]

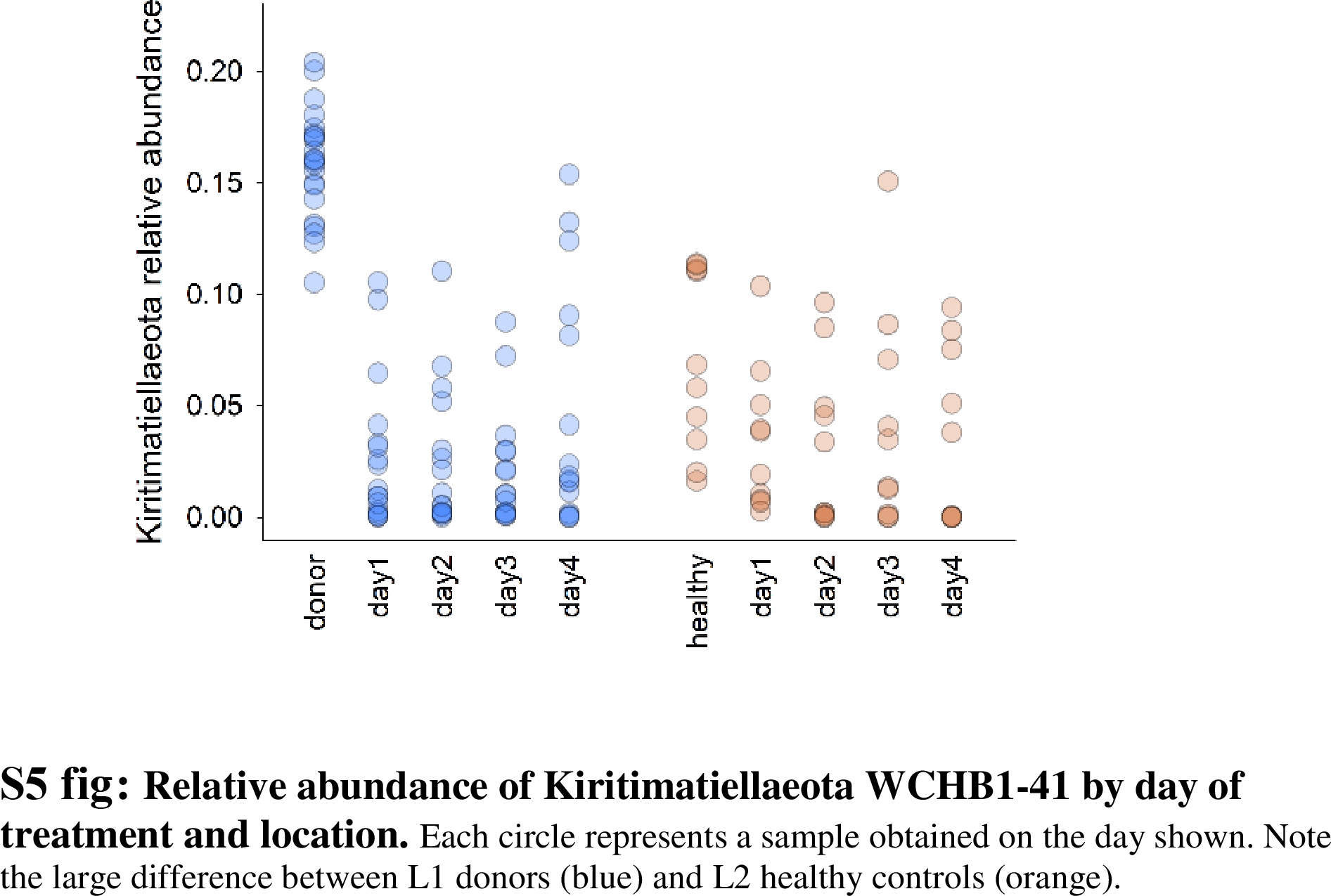

Supplement: S5 Fig — Each circle represents a sample obtained on the day shown. Note the large difference between L1 donors (blue) and L2 healthy controls (orange). (TIF) [file pone.0244381.s005.tif]

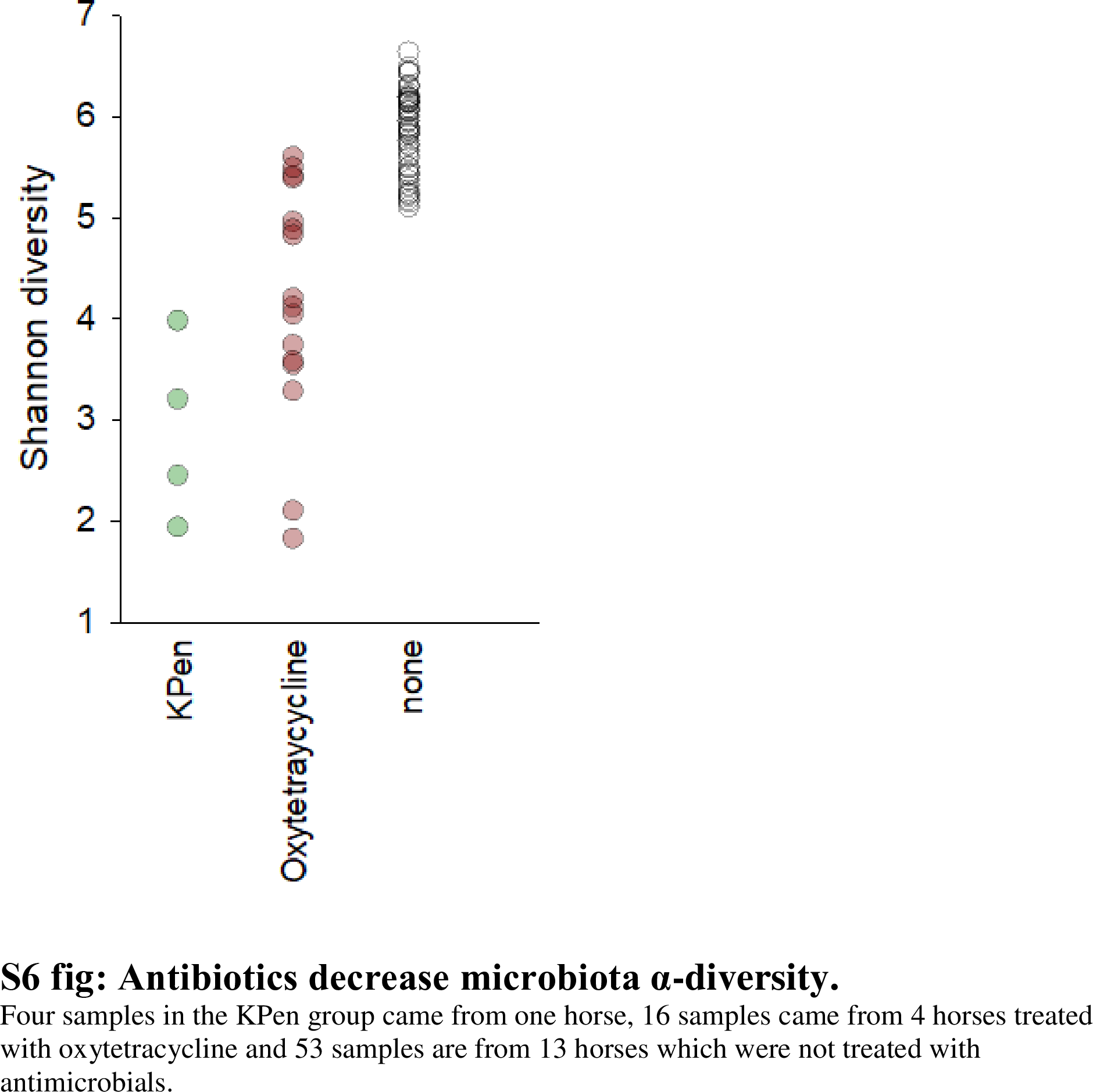

Supplement: S6 Fig — Four samples in the KPen group came from one horse, 16 samples came from 4 horses treated with oxytetracycline and 53 samples are from 13 horses which were not treated with antimicrobials. (TIF) [file pone.0244381.s006.tif]
