# Supplementary material for: Assessment of clinical and microbiota responses to fecal microbial transplantation in adult horses with diarrhea
Source: PLoS One. 2021 Jan 14;16(1):e0244381. doi: 10.1371/journal.pone.0244381 (PMC7808643; doi:10.1371/journal.pone.0244381)
Supplement: S1 Table — (DOCX) [file pone.0244381.s007.docx]

| **S1 Table. Procedure and sample collection timeline** | | | | | | | |
| --- | --- | --- | --- | --- | --- | --- | --- |
| Animal Group | Number of horses | Location* | Study Days: | 1 | 2 | 3 | 4 |
| Colitis Group receiving FMT | 12 | 1 | FMT  Physical examination  Feces collection  16S amplicon sequencing | x  x  x  x | x  x  x  x | x  x  x  x | x  x  x |
| Healthy FMT Donor Horses | 3 | 1 + N1 | Feces collection  16S amplicon sequencing | x  x | x  x | x  x |  |
| Healthy Controls | 30 | N1-N4 | Physical examination | x** |  |  |  |
|  |  |  | Feces collection | x |  |  |  |
|  |  |  | 16s amplicon sequencing | x |  |  |  |
| Colitis Group without FMT | 10 | 2 | Physical examination  Feces collection  16S amplicon sequencing | x  x  x | x  x  x | x  x  x | x  x  x |
| Healthy Controls | 10 | 2 | Physical examination  Feces collection | x  x |  |  |  |
|  |  |  | 16S amplicon sequencing | x |  |  |  |
| *Location1: Hospital where horses with colitis received FMT; Location 2: Hospital where horses with colitis did not receive FMT: **Healthy Controls from or in the regions surrounding L1 were sampled on two occasions, two weeks apart | | | | | | | |
