# Supplementary material for: Assessment of clinical and microbiota responses to fecal microbial transplantation in adult horses with diarrhea
Source: PLoS One. 2021 Jan 14;16(1):e0244381. doi: 10.1371/journal.pone.0244381 (PMC7808643; doi:10.1371/journal.pone.0244381)
Supplement: S2 Table — (DOCX) [file pone.0244381.s008.docx]

**S2 Table: Historical information of horses with diarrhea (colitis) at Location 1 receiving FMT**

| Patient ID | Age  (years) | Breed | Gender | BCS (1-9) | Presenting Complaint | Duration of diarrhea prior to enrollment (hours unless specified) | Duration of diarrhea following enrollment (hours) | Outcome |
| --- | --- | --- | --- | --- | --- | --- | --- | --- |
| RC | 22 | Paint | Gelding | 6 | Abdominal pain; Hypercreatinemia | 24 | 32 | Discharged |
| GH | 25.5 | Welsh Pony | Mare | 4 | Colitis | > 5 years | 111 | Discharged |
| RH | 9.5 | Warmblood | Gelding | 7 | Abdominal pain | 7 | 90 | Discharged |
| TT | 21 | Thoroughbred | Gelding | 6 | Abdominal pain; Fever | 24 | 90 | Discharged |
| JG | 26 | Quarter Horse | Gelding | 6 | Abdominal pain; History of chronic diarrhea | > 5 years | 65 | Discharged |
| CM | 22 | Irish Draft Horse | Mare | 9 | Colitis | 48 | 74 | Discharged |
| ZD | 7.5 | Warmblood | Gelding | 5 | Abdominal pain | 4 | 35 | Discharged |
| FB | 10 | Thoroughbred | Mare | 5 | Colitis; Endometritis | 2 | 111 | Discharged |
| AK | 13.5 | Warmblood | Gelding | 2 | Colitis | 24 | 182 | Discharged |
| JW | 19 | Warmblood | Gelding | 7 | Abdominal pain | 20 | 58 | Discharged |
| RR | 12 | Draft-Cross | Mare | 5 | Abdominal pain | 36 | 34 | Discharged |
| DH | 20 | Thoroughbred | Gelding | 4 | Abdominal pain | 5 | 14 | Discharged |

Body Condition Score, BCS: 1 = Emaciated, 5 = Ideal, 9 = Obese
