# Supplementary material for: Assessment of clinical and microbiota responses to fecal microbial transplantation in adult horses with diarrhea
Source: PLoS One. 2021 Jan 14;16(1):e0244381. doi: 10.1371/journal.pone.0244381 (PMC7808643; doi:10.1371/journal.pone.0244381)
Supplement: S3 Table — (DOCX) [file pone.0244381.s009.docx]

**S3 Table: Historical information of horses with diarrhea (colitis) at Location 2 not receiving FMT**

| Patient ID | Age  (years) | Breed | Gender | BCS (1-9) |  | Presenting Complaint | Duration of diarrhea prior to enrollment (hours) | Duration of diarrhea following enrollment (hours) | Outcome |
| --- | --- | --- | --- | --- | --- | --- | --- | --- | --- |
| VS | 29 | Saddlebred | Gelding | 1 |  | Colitis | 96 | 64 | Discharged |
| HW | 15 | Paso Fino-Cross | Gelding | 5 |  | Colitis | 96 | 24 | Discharged |
| DC | 16 | Thoroughbred | Gelding | 5 |  | Abdominal pain | 6 | 288 | Euthanized |
| PL | 2 | Quarter Horse | Mare | 5 |  | Colitis | 96 | 72 | Discharged |
| LC | 6 | Draft-cross | Mare | 7 |  | Abdominal pain | 24 | 72 | Discharged |
| NE | 10 | Quarter Horse | Mare | 5 |  | Colitis | 24 | 84 | Discharged |
| TM | 2 | Appaloosa | Gelding | 6 |  | Lethargy; Fever | 1 | 96 | Euthanized |
| MB | 25 | Morgan | Gelding | 5 |  | Abdominal pain | 11 | 24 | Discharged |
| MJ | 14 | Draft-cross | Mare | 6 |  | Colitis | 120 | 264 | Discharged |
| BW | 9 | Paint-cross | Gelding | 5 |  | Colitis | 24 | 90 | Discharged |

Body Condition Score, BCS: 1 = Emaciated, 5 = Ideal, 9 = Obese
