# Supplementary material for: Assessment of clinical and microbiota responses to fecal microbial transplantation in adult horses with diarrhea
Source: PLoS One. 2021 Jan 14;16(1):e0244381. doi: 10.1371/journal.pone.0244381 (PMC7808643; doi:10.1371/journal.pone.0244381)
Supplement: S4 Table — (DOCX) [file pone.0244381.s010.docx]

**S4 Table: Clinical enrollment parameters (Mean +/- Std Dev) of horses with colitis**

| Enrollment Clinical Variable | Location 1 | Location 2 | P value |
| --- | --- | --- | --- |
| Heart Rate (bpm)* | 37 +/- 8 | 50 +/- 24 | 0.017 |
| Respiratory Rate (brpm)* | 16 +/- 8 | 12 +/- 6 | 0.228 |
| Temperature (°F) | 99.9 +/- 0.55 | 100.3 +/- 1.8 | 0.532 |
| PCV (%) | 35.08 +/- 5.6 | 42.9 +/- 10.0 | 0.031 |
| TS (g/dL) | 6.38 +/- 0.76 | 5.66 +/- 1.5 | 0.184 |
| Lactate (mmol/L) | 0.76 +/- 0.3 | 1.73 +/- 0.8 | 0.004 |
| Pre-enrollment diarrhea duration (hours)* | 24 +/- 34.8 | 24 +/- 86.3 | 0.821 |
| Enrollment diarrhea score | 5 +/- 1.0 | 3.5 +/- 1.5 | 0.05 |

Heart rate, bpm: beats per minute; Respiratory rate, brpm: breaths per minute; Temperature, degrees Fahrenheit; PCV: Packed cell volume; TS: total serum solids; Enrollment diarrhea score: 0 -9; Location 1: University Hospital on the East Coast; Location 2: University Hospital on the West Coast; *Median +/- Interquartile range
