# Supplementary material for: Assessment of clinical and microbiota responses to fecal microbial transplantation in adult horses with diarrhea
Source: PLoS One. 2021 Jan 14;16(1):e0244381. doi: 10.1371/journal.pone.0244381 (PMC7808643; doi:10.1371/journal.pone.0244381)
Supplement: S5 Table — (DOCX) [file pone.0244381.s011.docx]

**S5 Table: Final clinical parameters (Mean +/- Std Dev) of horses with colitis upon study completion**

| Clinical Variable | Location 1 | Location 2 | P value |
| --- | --- | --- | --- |
| Heart Rate (bpm) | 36.3 +/- 5.5 | 43 +/- 12.4 | 0.083 |
| Respiratory Rate (brpm)* | 12 +/- 4 | 16 +/- 4 | 0.497 |
| Temperature (°F) | 99.5 +/- 0.76 | 100.0 +/- 1.2 | 0.227 |
| PCV (%)* | 36.5 +/- 3.4 | 36 +/- 6 | 0.722 |
| TS (g/dL) | 6.5 +/- 0.84 | 5.8 +/- 1.3 | 0.078 |
| Lactate (mmol/L) | 0.82 +/- 0.3 | 1.4 +/- 0.4 | 0.002 |
| Post-enrollment diarrhea duration (hours) | 69.5 +/- 71.5 | 78 +/- 84 | 0.674 |
| Final Diarrhea Score | 0 +/- 1.75 | 1 +/- 3 | 0.098 |

Heart rate, bpm: beats per minute; Respiratory rate, brpm: breaths per minute; Temperature, degrees Fahrenheit; PCV: Packed cell volume; TS: L1 total serum solids, L2 plasma total solids; Enrollment diarrhea score: 0 -9; Location 1: University Hospital on the East Coast; Location 2: University Hospital on the West Coast; *Median +/- Interquartile range
