# Supplementary material for: Assessment of clinical and microbiota responses to fecal microbial transplantation in adult horses with diarrhea
Source: PLoS One. 2021 Jan 14;16(1):e0244381. doi: 10.1371/journal.pone.0244381 (PMC7808643; doi:10.1371/journal.pone.0244381)
Supplement: S6 Table — (DOCX) [file pone.0244381.s012.docx]

**S6 Table: Median Diarrhea Scores (+/- IQR) of horses with colitis over time**

| Median Diarrhea Score | Location 1 | Location 2 | P value |
| --- | --- | --- | --- |
| Enrollment | 5 +/- 1.0 | 3.5 +/- 1.5 | 0.05 |
| Day 1 | 4.75 +/- 2.0 | 3.75 +/- 2.38 | 0.254 |
| Day 2 | 3.25 +/- 4.0 | 3.0 +/- 4.0 | 0.771 |
| Day 3 | 1.75 +/- 3.38 | 3.0 +/- 3.38 | 0.346 |
| Final | 0 +/- 1.75 | 1 +/- 3 | 0.098 |

Manure scoring system: 0: Normal, firm but moist balls of manure; 1: Formed balls of manure that lose their form upon reaching the ground; 2: Pudding-consistency manure that still holds some shape; 3: Pudding-consistency manure that spreads out upon reaching the ground; 4: Watery manure with some formed pieces; 5: Watery manure without formed pieces.
