# Supplementary material for: Assessment of clinical and microbiota responses to fecal microbial transplantation in adult horses with diarrhea
Source: PLoS One. 2021 Jan 14;16(1):e0244381. doi: 10.1371/journal.pone.0244381 (PMC7808643; doi:10.1371/journal.pone.0244381)
Supplement: S7 Table — (DOCX) [file pone.0244381.s013.docx]

**S7 Table: Healthy Donors and Control Horses’ phenotype, diet, and location (L)**

| ID | Location | Age (years) | Sex | BCS | Diet - Hay | Pellet Type | Diet – Concentrate (kg per day) | Pasture turnout (hours) |
| --- | --- | --- | --- | --- | --- | --- | --- | --- |
| Donor 1 | N1 | 6 | Gelding | 7 | 2% BW 1^st^ Cut Grass Hay | 30% Protein, 5% Fat | 0.6 | 14 |
| Donor 2 | L1 | 12 | Mare | 5.5 | 1-1.5% BW 2^nd^ Cut Grass Hay | 14% Protein, 7% Fat | 1.4 | 3 |
| Donor 3 | L1 | 10 | Gelding | 6 | 1-1.5% BW 2^nd^ Cut Grass Hay | 14% Protein, 7% Fat | 1.8 | 3 |
| C 1-1 | N1 | 6 | Gelding | 7 | 2% BW 1^st^ Cut Grass Hay | 30% Protein, 5% Fat | 0.6 | 14 |
| C 2-1 | N1 | 22 | Gelding | 7 | 2% BW 1^st^ Cut Grass Hay | 30% Protein, 5% Fat | 0.6 | 14 |
| C 3-1 | N2 | 11 | Gelding | 6.5 | 2% BW 1^st^ Cut Grass Hay | 14% Protein, 7% Fat | 1.8 | 3 |
| C 4-1 | N2 | 20 | Gelding | 5 | 1.5-2% BW 1^st^ Cut Grass Hay | 14% Protein, 7% Fat | 1.4 | 6 |
| C 5-1 | N2 | 12 | Mare | 5.5 | 2% BW 1st Cut Grass Hay | 14% Protein, 7% Fat | 1.4 | 6 |
| C 6-1 | N2 | 25.5 | Mare | 4 | 1.5-2% BW 1st Cut Grass Hay | 14% Protein, 7% Fat | 1.4 | 6 |
| C 7-1 | N2 | 10 | Gelding | 6 | 2% BW 1^st^ Cut Grass Hay | 30% Protein, 5% Fat | 0.3 | 14 |
| C 8-1 | N2 | 25 | Gelding | 6 | 1.5-2% BW 1st Cut Grass Hay | 14% Protein, 7% Fat | 1.4 | 3 |
| C 9-1 | N3 | 2 | Mare | 6 | 2-2.5% BW 1^st^ Cut Grass Hay | 13% Protein, 8% Fat | 2.7 | 0 |
| C 10-1 | N3 | 20 | Mare | 5 | 2-2.5% BW 1^st^ Cut Grass Hay | 13% Protein, 8% Fat | 1.8 | 0 |
| C 11-1 | N3 | 2 | Mare | 7 | 2-2.5% BW 1^st^ Cut Grass Hay | 13% Protein, 8% Fat | 2.7 | 0 |
| C 12-1 | N3 | 22 | Mare | 6 | 2-2.5% BW 1^st^ Cut Grass Hay | 13% Protein, 8% Fat | 3.6 | 0 |
| C 13-1 | N3 | 7 | Mare | 7 | 2-2.5% BW 1^st^ Cut Grass Hay | 13% Protein, 8% Fat | 2.7 | 0 |
| C 14-1 | N3 | 25 | Mare | 7 | 2-2.5% BW 1^st^ Cut Grass Hay | 13% Protein, 8% Fat | 2.7 | 0 |
| C 15-1 | N3 | 2 | Mare | 7 | 2-2.5% BW 1^st^ Cut Grass Hay | 13% Protein, 8% Fat | 2.7 | 0 |
| C 16-1 | N3 | 20 | Mare | 8 | 2-2.5% BW 1^st^ Cut Grass Hay | 13% Protein, 8% Fat | 2.7 | 0 |
| C 17-1 | N3 | 23 | Mare | 5 | 2-2.5% BW 1^st^ Cut Grass Hay | 13% Protein, 8% Fat | 4.5 | 0 |
| C 18-1 | N3 | 2 | Mare | 5 | 2-2.5% BW 1^st^ Cut Grass Hay | 13% Protein, 8% Fat | 2.7 | 0 |
| C 19-1 | N3 | 21 | Mare | 4.5 | 2-2.5% BW 1^st^ Cut Grass Hay | 13% Protein, 8% Fat | 3.6 | 0 |
| C 20-1 | N3 | 10 | Mare | 5 | 2-2.5% BW 1^st^ Cut Grass Hay | 13% Protein, 8% Fat | 4.5 | 0 |
| C 21-1 | N3 | 24 | Mare | 4 | 2-2.5% BW 1^st^ Cut Grass Hay | 13% Protein, 8% Fat | 2.7 | 0 |
| C 22-1 | N3 | 4 | Gelding | 5 | 2-2.5% BW 1^st^ Cut Grass Hay | 13% Protein, 8% Fat | 2.7 | 0 |
| C 23-1 | N4 | 10 | Mare | 8 | 2% BW 2^nd^ Cut Grass Hay | 30% Protein, 3% Fat | 0.5 | 6 |
| C 24-1 | N4 | 23 | Mare | 8.5 | 2% BW 2^nd^ Cut Grass Hay | 30% Protein, 3% Fat | 0.5 | 6 |
| C 25-1 | N4 | 5 | Mare | 8 | 2% BW 1^st^ Cut Grass Hay | 30% Protein, 3% Fat | 0.5 | 6 |
| C 26-1 | N4 | 23 | Mare | 8.5 | 2% BW 1^st^ Cut Grass Hay | 30% Protein, 3% Fat | 0.5 | 6 |
| C 27-1 | N4 | 10 | Mare | 8 | 2% BW 1^st^ Cut Grass Hay | 30% Protein, 3% Fat | 0.5 | 6 |
| C 29-1 | N4 | 22 | Mare | 9 | 2% BW 1^st^ Cut Grass Hay | 30% Protein, 3% Fat | 0.5 | 6 |
| C 28-1 | N4 | 7 | Gelding | 6 | 2% BW 1^st^ Cut Grass Hay | 14% Protein, 10% Fat | 1.7 | 24 |
| C 30-1 | N4 | 23 | Gelding | 5 | 2% BW 1^st^ Cut Grass Hay | 14% Protein, 10% Fat | 1.7 | 24 |
| C 1-2 | L2 | 3 | Mare | 7 | 2% BW 2^nd^ Cut Grass Hay | None | None | 9 |
| C 2-2 | L2 | 20 | Mare | 6 | 2% BW 2^nd^ Cut Grass Hay | None | None | 9 |
| C 3-2 | L2 | 18 | Mare | 6 | 2% BW 2^nd^ Cut Grass Hay | None | None | 9 |
| C 4-2 | L2 | 19 | Mare | 6 | 2% BW 2^nd^ Cut Grass Hay | None | None | 9 |
| C 5-2 | L2 | 20 | Mare | 6 | 2% BW 2^nd^ Cut Grass Hay | None | None | 9 |
| C 6-2 | L2 | 23 | Mare | 5 | 2% BW 2^nd^ Cut Grass Hay | 14% Protein, 8% Fat | 4 | 9 |
| C 7-2 | L2 | 20 | Mare | 6 | 2% BW 2^nd^ Cut Grass Hay | None | None | 9 |
| C 8-2 | L2 | 13 | Mare | 6 | 2% BW 2^nd^ Cut Grass Hay | None | None | 9 |
| C 9-2 | L2 | 18 | Mare | 6 | 2% BW 2^nd^ Cut Grass Hay | None | None | 9 |
| C 10-2 | L2 | 23 | Mare | 5 | 2% BW 2^nd^ Cut Grass Hay | None | None | 9 |
