# Supplementary material for: Assessment of clinical and microbiota responses to fecal microbial transplantation in adult horses with diarrhea
Source: PLoS One. 2021 Jan 14;16(1):e0244381. doi: 10.1371/journal.pone.0244381 (PMC7808643; doi:10.1371/journal.pone.0244381)
Supplement: S9 Table — (DOCX) [file pone.0244381.s015.docx]

**S9 Table: Classification of OTUs significantly different in relative abundance between L2 healthy and L2 diarrheic horses**

|  | | | | | | |
| --- | --- | --- | --- | --- | --- | --- |
| Taxon | healthy | colitis | expected | | (colitis+healthy)/  expected | |
| Bacteria_unclassified | 35 | 3 | | 291 | | 0.13 |
| Bacteroidetes | 224 | 14 | | 1124 | | 0.21 |
| Cyanobacteria | 10 | 0 | | 34 | | 0.29 |
| Epsilonbacteraeota | 0 | 1 | | 3 | | 0.33 |
| Fibrobacteres | 9 | 0 | | 30 | | 0.30 |
| Firmicutes | 132 | 10 | | 1315 | | 0.11 |
| Kiritimatiellaeota | 47 | 0 | | 296 | | 0.16 |
| Lentisphaerae | 4 | 0 | | 28 | | 0.14 |
| Proteobacteria | 8 | 2 | | 86 | | 0.12 |
| Spirochaetes | 16 | 1 | | 124 | | 0.14 |
| unclassified | 1 | 0 | | 0 | |  |
| * See Table 3 footnotes | | | | | | |
