# Supplementary material for: Assessment of clinical and microbiota responses to fecal microbial transplantation in adult horses with diarrhea
Source: PLoS One. 2021 Jan 14;16(1):e0244381. doi: 10.1371/journal.pone.0244381 (PMC7808643; doi:10.1371/journal.pone.0244381)
Supplement: S10 Table — (DOCX) [file pone.0244381.s016.docx]

|  | | | | |
| --- | --- | --- | --- | --- |
| Taxon | L1 | L2 | expected | (L1+L2)/  expected |
| Bacteroidetes | 12 | 6 | 1124 | 0.02 |
| Firmicutes | 7 | 7 | 1315 | 0.01 |
| Fibrobacteres | 1 | 0 | 30 | 0.03 |
| Verrucomicrobia | 1 | 0 | 8 | 0.13 |
| * See Table 3 footnotes | | | | |

**S10 Tables: Classification of OTUs significantly different in relative abundance between diseased L1 and L2 horses**
