# Supplementary material for: Assessment of clinical and microbiota responses to fecal microbial transplantation in adult horses with diarrhea
Source: PLoS One. 2021 Jan 14;16(1):e0244381. doi: 10.1371/journal.pone.0244381 (PMC7808643; doi:10.1371/journal.pone.0244381)
Supplement: S11 Table — (DOCX) [file pone.0244381.s017.docx]

**S11 Table: Classification of OTUs significantly different in relative abundance between healthy L1 donors and healthy L2 horses**

|  | | | | |
| --- | --- | --- | --- | --- |
| Taxon | L1 | L2 | expected | (L1+L2)/  expected |
| Bacteria_unclassified | 10 | 18 | 285 | 0.10 |
| Bacteroidetes | 48 | 131 | 1124 | 0.16 |
| Cyanobacteria | 0 | 2 | 34 | 0.06 |
| Fibrobacteres | 1 | 5 | 30 | 0.20 |
| Firmicutes | 26 | 54 | 1314 | 0.06 |
| Kiritimatiellaeota | 57 | 6 | 276 | 0.21 |
| Lentisphaerae | 2 | 1 | 28 | 0.11 |
| Proteobacteria | 2 | 3 | 86 | 0.06 |
| Spirochaetes | 7 | 11 | 124 | 0.15 |
| Tenericutes | 1 | 0 | 26 | 0.04 |
| * See Table 3 footnotes | | | | |
